# The transcriptional profile of coronary arteritis in Kawasaki disease

**DOI:** 10.1186/s12864-015-2323-5

**Published:** 2015-12-18

**Authors:** Anne H. Rowley, Kristine M. Wylie, Kwang-Youn A. Kim, Adam J. Pink, Amy Yang, Rebecca Reindel, Susan C. Baker, Stanford T. Shulman, Jan M. Orenstein, Mark W. Lingen, George M. Weinstock, Todd N. Wylie

**Affiliations:** Department of Pediatrics, Northwestern University Feinberg School of Medicine, 310 E Superior Street, Morton 4-685B, Chicago, IL 60611 USA; Department of Microbiology and Immunology, Northwestern University Feinberg School of Medicine, Chicago, IL USA; Department of Preventive Medicine, Northwestern University Feinberg School of Medicine, Chicago, IL USA; Ann & Robert H. Lurie Children’s Hospital of Chicago, Chicago, IL USA; Department of Pediatrics, Washington University School of Medicine, Saint Louis, MO USA; The McDonnell Genome Institute at Washington University, Washington University School of Medicine, Saint Louis, MO USA; Department of Microbiology/Immunology, Loyola University Stritch School of Medicine, Maywood, IL USA; Department of Pathology, George Washington University School of Medicine, Washington, DC USA; Department of Pathology, University of Chicago Pritzker School of Medicine, Chicago, IL USA; Present address: AbbVie, Inc, North Chicago, IL USA; Present address: The Jackson Laboratory for Genomic Medicine, Farmington, CT USA

**Keywords:** Kawasaki disease, Coronary artery aneurysm, Arteritis, Childhood, Innate immune response, Acquired immune response

## Abstract

**Background:**

Kawasaki Disease (KD) can cause potentially life-threatening coronary arteritis in young children, and has a likely infectious etiology. Transcriptome profiling is a powerful approach to investigate gene expression in diseased tissues. RNA sequencing of KD coronary arteries could elucidate the etiology and the host response, with the potential to improve KD diagnosis and/or treatment.

**Methods:**

Deep RNA sequencing was performed on KD (*n* = 8) and childhood control (*n* = 7) coronary artery tissues, revealing 1074 differentially expressed mRNAs. Non-human RNA sequences were subjected to a microbial discovery bioinformatics platform, and microbial sequences were analyzed by Metastats for association with KD.

**Results:**

T lymphocyte activation, antigen presentation, immunoglobulin production, and type I interferon response were significantly upregulated in KD arteritis, while the tumor necrosis factor α pathway was not differentially expressed. Transcripts from known infectious agents were not specifically associated with KD coronary arteritis.

**Conclusions:**

The immune transcriptional profile in KD coronary artery tissues has features of an antiviral immune response such as activated cytotoxic T lymphocyte and type I interferon-induced gene upregulation. These results provide new insights into the pathogenesis of KD arteritis that can guide selection of new immunomodulatory therapies for high-risk KD patients, and provide direction for future etiologic studies.

**Electronic supplementary material:**

The online version of this article (doi:10.1186/s12864-015-2323-5) contains supplementary material, which is available to authorized users.

## Background

Transcriptome profiling of infected tissues, or “dual RNA-sequencing” allows for unbiased simultaneous gene expression evaluation of pathogen and host [1]. Kawasaki Disease (KD) is an acute febrile illness of young childhood that can cause medium-sized muscular arteritis, most critically affecting the coronary arteries, and a large body of clinical, epidemiologic, and experimental evidence points to an infectious cause [2, 3]. Severely affected infants and young children develop coronary artery aneurysms and are at risk for myocardial infarction and sudden death [3]. It has been difficult to improve diagnosis and treatment of KD because of a lack of understanding of the etiology and pathogenesis. The purpose of this study was to identify specific cellular pathways and infectious agents in KD coronary arteritis by transcriptome profiling, to elucidate the pathogenesis of the disease.

## Methods

### Patients and controls

The study was approved by the Institutional Review Board of The Ann & Robert H. Lurie Children’s Hospital of Chicago. The KD cases occurred widely throughout the United States over the last three decades. Informed consent was obtained at the primary institution in five cases; in the remaining three KD cases and all the control cases, tissues were archival and de-identified. Six KD patients were male and two were female. Five were Caucasian, one Hispanic, one African-American, and one of unknown ethnicity. The mean age of the KD patients was 15 months with a median age of 7 months. Eight control cases were male and 3 were female. The mean age of the control patients was 15 months with a median age of 5 months. Data deposited at Gene Expression Omnibus, National Center for Biotechnology Information (GEO) cannot be potentially used to re-identify individuals from the study. Clinical information on the KD patients is given in Table 1. All patients had coronary artery abnormalities and all were fatal cases except for KD3, who underwent heart transplant; the coronary artery light and electron microscopic findings for KD patients 1–3 and 5–8 are described in our pathologic study [4] (Tables 1 and 2). Additional information for KD patient 4 is provided in Additional file 1: Supplemental Methods. Childhood controls had normal coronary artery histology; their diagnoses are given in Table 2. Three of the KD patients did not receive any therapy between fever onset and death/transplant and one received only aspirin and dipyridamole (KD1-4, “untreated” group). One patient received IGIV alone, two received IGIV and corticosteroid, and one patient received IGIV, corticosteroid, and infliximab (KD5-8, “treated” group).

**Table 1:** Clinical data on Kawasaki disease children whose coronary artery tissues were tested in this study

Case #	Time since onset	KD therapy	Category	Pathology study case # [4]	RNA tested by
KD1	2.5 weeks	None	Untreated	4	HTS, PCR
KD2	4 weeks	ASA, dipyridamole	Untreated	16	HTS
KD3	5 months	None	Untreated	26	HTS, PCR
KD4	7 months	None	Untreated	NA	HTS, PCR
KD5	3.5 weeks	IGIV, ASA, steroid	Treated	11	HTS, PCR
KD6	4 weeks	IGIV, ASA, steroid, infliximab	Treated	13	HTS, PCR
KD7	3 weeks	IGIV, ASA	Treated	7	HTS, PCR
KD8	5 weeks	IGIV, ASA, steroid	Treated	18	HTS

*HTS* high-throughput RNA sequencing, *PCR* real-time reverse transcriptase PCR, *ASA* aspirin

**Table 2:** Clinical data on control children whose coronary artery tissues were tested in this study

Control #	Diagnosis	RNA tested by
C1	Enterobacter sepsis, pulmonary hemorrhage, neurologic devastation from herpes simplex virus encephalitis	HTS
C2	Pneumococcal meningitis, disseminated intravascular coagulation	HTS, PCR
C3	Prematurity, neurologic devastation secondary to Serratia meningitis, chronic lung disease	HTS
C4	Meconium aspiration, pulmonary hemorrhage	HTS
C5	Developmental delay, seizures, fever	HTS
C6	Prematurity, cerebral hemorrhage, bronchopulmonary dysplasia, and pneumonia	HTS, PCR
C7	Cholestasis, renal tubular acidosis, agenesis corpus callosum, dehydration	HTS
C8	Congenital diaphragmatic hernia, pulmonary hypoplasia	PCR
C9	Hypotonia, subdural and liver hematomas	PCR
C10	Congenital sacrococcygeal teratoma	PCR
C11	Hypoplastic left heart, respiratory syncytial virus infection	PCR

*HTS* high-throughput RNA sequencing, *PCR* real-time reverse transcriptase PCR

### RNA isolation and quality control analyses

RNA was isolated from formalin-fixed, paraffin-embedded KD and control tissue sections using the RNeasy kit designed for these tissues (Qiagen, Valencia, CA). RNA samples meeting the quality standards described in Additional file 1: Supplemental Methods were subjected to ribosomal RNA subtraction using the Ribo-Zero human rRNA subtraction kit (Epicentre, Madison, WI) prior to sequencing.

### High-throughput RNA sequencing

Preparation of cDNA libraries and Illumina HiSeq2000 RNA sequencing were performed at the University of Utah Microarray Core Facility (Salt Lake City, UT).

### Sequence alignment and normalization

For RNA sequencing analysis, TopHat (Bowtie version 2.1.0) [5] was used to align reads to the human reference GRCh37-lite (accession id: GCA_000001405.1), and HTseq version 0.6.1 in “union” mode [6] was used to determine read counts. DESeq was used for variance stabilizing normalization and determination of differential expression based on a model using the negative binomial distribution [7]. Additional information on alignments can be found in Additional file 1: Table S1.

### Pathways analysis

The data were analyzed using iReport™ (Ingenuity Systems, Redwood City, CA, www.ingenuity.com), with fold change of at least 1.5 and *q-*value of ≤ 0.05 as significance criteria for differentially expressed genes between KD patients and controls. The primary analysis was of CA gene expression in 8 KD patients compared to 7 childhood controls. Subgroup analyses were also performed for the CA transcriptome of 4 untreated KD patients compared to 7 controls, and 4 treated KD patients compared to 7 controls.

### Cluster and principal components analyses

Hierarchical cluster analysis with mean linkage function (hclust function in base R) was used to cluster the samples by expression profiles. Principal components analysis was run to estimate the variances explained by the first and second principal components (prcomp function in base R).

### Real-time PCR

Differential expression by real-time PCR were performed on KD and control coronary artery formalin-fixed, paraffin-embedded tissue RNAs using the comparative C_T_ method [8]. The differential expression was compared using variances estimated by empirical Bayes models. We controlled for false discovery rate to account for multiple testing under dependency using adjusted *p*-values [9].

### Microbial analyses

Viral sequences were identified based on nucleotide and translated amino acid sequence alignments as described [10]. Sequences from cellular microbes were identified using Real Time Genomics Map and Species programs, version 3.4.3 (Real Time Genomics, Hamilton, New Zealand, http://realtimegenomics.com) to align sequences against the Human Microbiome Project’s microbial reference genome database (http://hmpdacc.org/HMREFG/), and by classifying sequences with MetaPhlAn [11]. Metastats was used to test for sequences that were differentially abundant in KD cases and controls [12].

## Results

### Deep RNA sequencing of KD and pediatric control coronary arteries

**Fig. 1:**
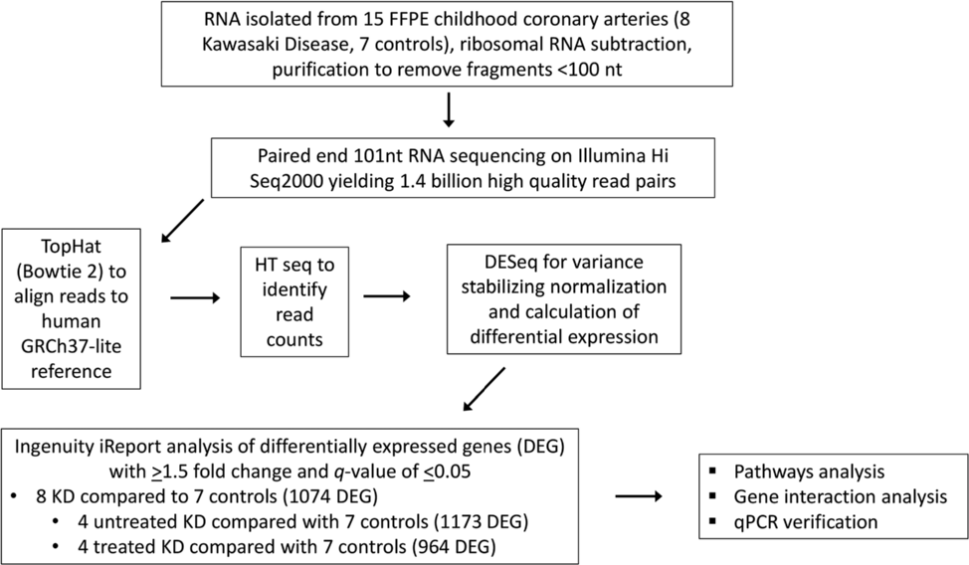
Workflow to determine differentially expressed molecular pathways in KD coronary arteries

**Fig. 2:**
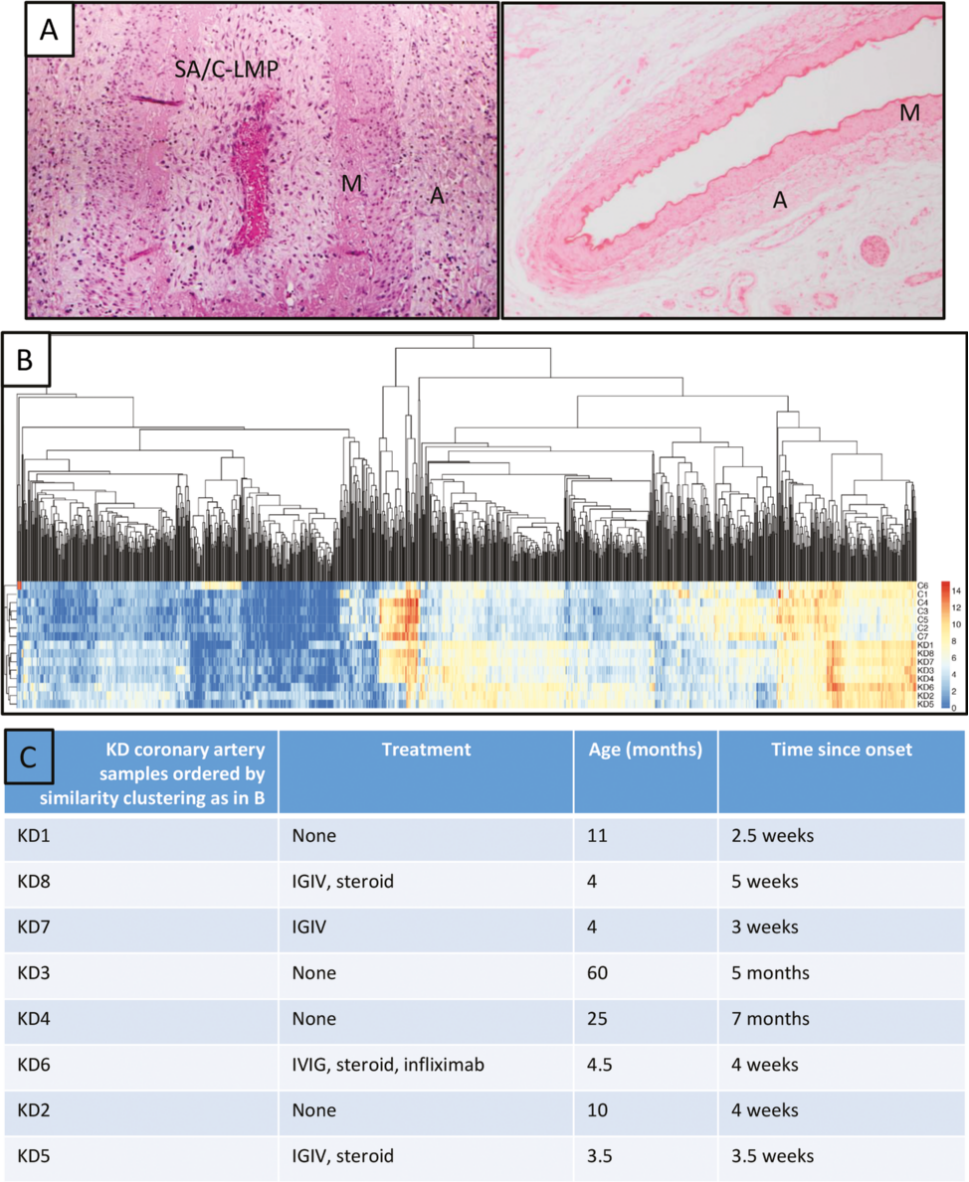
**a** Left, coronary artery tissue section from KD patient 6, demonstrating subacute chronic (SA/C) inflammation and luminal myofibroblastic proliferation (LMP) with resultant narrowing of the arterial lumen that is filled with blood. Right, coronary artery tissue section from control patient 4, demonstrating normal histology without inflammation. Hematoxylin and eosin stains, M = media, A = adventitia; **b** Heatmap demonstrating differential gene expression in KD and control coronary artery tissues, with low expression in blue and high expression in red; **c** KD coronary artery gene expression does not cluster by treatment, patient age, or time since onset of KD. The lack of clustering of gene expression by treatment is likely due to persistent arteritis in the treated patients despite therapy

To evaluate the transcriptome of KD arteritis, we compared the RNA expression of KD coronary artery tissues with those of non-inflamed coronary artery tissues from pediatric patients with non-KD illnesses as controls. The analysis pathway is shown in Fig. 1. The pathologic features of the coronary arteries in the KD patients are described in our prior study [4]; all showed subacute/chronic arteritis and luminal myofibroblastic proliferation (Fig. 2a). All primary data can be accessed at NCBI GEO GSE64486. Sequencing yielded a total of 40–120 million RNA sequencing reads/sample. About 50–90 % of the reads for each tissue sample mapped to the human genome. DEseq analysis yielded 1074 genes that were differentially expressed in 8 KD compared with 7 childhood control coronary arteries (Fig. 2b and c and Additional file 1: Table S2). Strikingly, heat map analysis of differentially expressed genes in KD and control coronary artery tissues showed that gene expression in KD patients did not cluster by age, time since onset of KD, or treatment, supporting the analysis of the 8 KD tissue samples together (Fig. 2b and c). The lack of clustering by time since onset of KD is consistent with our pathologic study demonstrating persistence of subacute arteritis for months to years after the onset [4]. It is notable in Fig. 2b and c that the gene expression profiles of the two patients with the longest time interval since onset, KD3 and KD4, fall directly in the center of the KD patient group. The lack of clustering by treatment is likely explained by persistent arteritis in the treated patients despite therapy. Principal components analysis also demonstrated that gene expression profiles in treated and untreated KD patients were not distinguishable (Additional file 1: Figure S1).

### T lymphocyte activation and Type I interferon-induced genes are upregulated in KD coronary arteries

**Fig. 3:**
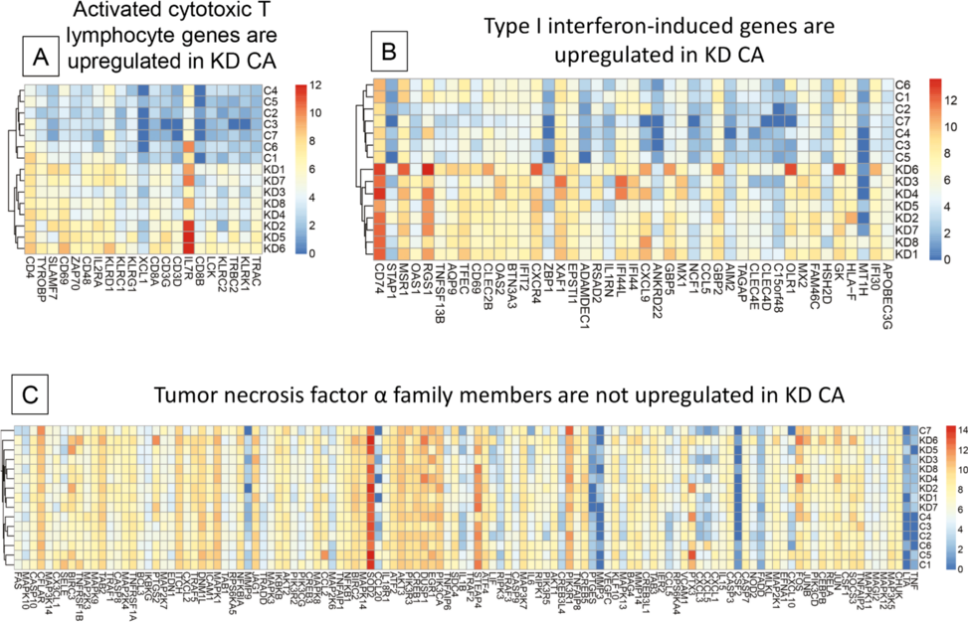
Expression levels of gene families in KD compared with control coronary arteries. **a** Upregulation of activated cytotoxic CD8 T lymphocyte genes in KD, **b** Upregulation of type I interferon-induced genes in KD, **c** Lack of differential expression of tumor necrosis factor α-induced genes in KD. Blue indicates low expression and red high expression

Pathways associated with activated T lymphocyte function were significantly upregulated in KD coronary arteries (Table 3, Additional file 1: Table S3). Activation of cytotoxic T lymphocyte genes is consistent with our prior report demonstrating CD8 protein expression in KD coronary arteries [13] (Fig. 3a). Although molecular pathways associated with natural killer cell signaling were identified as upregulated in KD coronary arteries (Table 3), there is marked overlap in gene expression by natural killer cells and activated cytotoxic CD8 T lymphocytes [14], and the lack of an antibody that can reliably distinguish these two cell types by immunohistochemistry did not allow us to determine whether natural killer cells were present in the coronary artery inflammatory infiltrate. In addition, many immunoglobulin genes were upregulated, consistent with our prior immunohistochemical studies showing immunoglobulin protein expression in the inflammatory infiltrate [15].

**Table 3:** Top 25 upregulated pathways in kawasaki disease coronary arteries

Upregulated pathways	# of differentially expressed genes	*p-*value
Primary immunodeficiency signaling	26	5.65E-21
Communication between innate and adaptive immune cells	30	1.08E-17
iCOS-iCOSL signaling in T helper cells	32	3.12E-17
Altered T cell and B cell signaling in rheumatoid arthritis	29	3.84E-17
B cell development	17	3.62E-14
CD28 signaling in T helper cells	28	1.35E-12
Role of NFAT in regulation of the immune response	33	6.43E-12
PKCθ signaling in T lymphocytes	27	8.5E-12
Autoimmune thyroid disease signaling	17	5.1E-11
Hematopoiesis from pluripotent stem cells	17	1.06E-10
T cell receptor signaling	23	1.38E-10
Antigen presentation pathway	14	6.89E-10
Calcium-induced T lymphocyte apoptosis	18	7.03E-10
Crosstalk between dendritic cells and natural killer cells	21	9.61E-10
Systemic lupus erythematosus signaling	34	1.8E-09
Allograft rejection signaling	20	3.16E-09
Graft-versus-host disease signaling	15	3.7E-09
T helper cell differentiation	18	4.41E-09
CTLA4 signaling in cytotoxic T lymphocytes	20	4.84E-09
Nur77 signaling in T lymphocytes	16	6.42E-09
Dendritic cell maturation	29	8.49E-09
Agranulocyte adhesion and diapedesis	29	2.97E-08
OX40 signaling pathway	19	3.48E-08
Natural killer cell signaling	21	5.32E-08
Hepatic fibrosis/Hepatic stellate cell activation	29	7.53E-08

Many type I interferon-stimulated genes [16–18] were upregulated (Table 4, Fig. 3b). This contrasts with previously reported findings in the peripheral blood of acute KD patients, in which interferon-stimulated gene expression was reported to be strikingly absent [19]. This result emphasizes the limitations of peripheral blood studies in determining gene expression changes in diseased target tissues.

**Table 4:** Type I interferon-stimulated genes differentially expressed in KD compared with childhood control coronary arteries

Gene	Fold change	*q-*value
ADAMDEC1	20.5	2.6e-18
AIM2	3.7	0.023
ANKRD22	10.2	1.9e-07
APOBEC3G	3.5	0.023
AQP9	4.9	1.7e-06
BTN3A3	3.5	0.020
C15orf48	4.0	0.0004
CCL5	6.5	1.1e-05
CD69	10.3	2.6e-09
CD74	7.7	3.5e-05
CLEC2B	3.0	0.006
CLEC4D	3.4	0.029
CLEC4E	3.9	0.011
CXCL9	48.0	1.2e-14
CXCR4	14.7	2.9e-12
EPSTI1	4.9	0.002
FAM46C	5.3	0.001
GBP2	4.3	0.0005
GBP5	14.7	8.8e-14
GK	2.6	0.0008
HLA-F	2.8	0.007
HSH2D	2.9	0.044
IFI30	4.6	0.0002
IFI44	3.3	0.022
IFI44L	4.2	4.6e-06
IFIT2	3.3	0.029
IL1RN	2.8	0.020
MSR1	5.8	0.002
MT1H	1.7	0.0009
MX1	4.2	0.0002
MX2	4.7	0.0005
NCF1	4.1	0.004
OAS1	4.3	0.008
OAS2	5.0	0.0005
OLR1	42.2	7.6e-19
RGS1	17.0	1.3e-10
RSAD2	4.1	0.002
STAP1	7.4	1.3e-07
TAGAP	6.2	0.0001
TFEC	7.4	0.0009
TNFSF13B	4.2	0.010
XAF1	3.3	0.005
ZBP1	5.6	0.002
AGPAT9	−2.4	0.0008
CES1	−4.1	4.0e-05
FNDC4	−2.0	0.006
MT1M	−2.5	0.029
SAA1	−5.3	6.4e-14

### Antigen presentation and dendritic cell pathways are activated in KD arteritis

Pathways associated with dendritic cells and antigen presentation were significantly upregulated (Table 3). The activating Fc receptor genes FCGR2A, FCGR2C, FCGR3A, and FCGR3B, commonly expressed on dendritic cells, were upregulated, while the inhibitory receptor FCGR2B and the macrophage marker FCGR1 were not differentially expressed.

### Macrophage and neutrophil-specific gene expression are not prominent

Genes commonly expressed by monocyte/macrophages [20] were not differentially expressed, such as CD68, FCGR1A, CSF1R, CD163, and CD14. This contrasts with a view of KD arteritis as a primarily macrophage-mediated process [21]. Neutrophil-associated genes were not significantly altered. Eosinophils are a component of the unique subacute/chronic arteritis infiltrate, and SIGLEC8, which is selectively expressed by eosinophils and mast cells [22], was upregulated.

### Pattern recognition receptor genes are differentially expressed in KD arteritis

Pattern recognition receptor genes and Toll-like receptor signaling pathways were differentially expressed (Table 3). Many C-type lectin and cytosolic pattern-recognition receptors were differentially expressed (Additional file 1: Table S2). The most highly upregulated gene in the Toll-like receptor family was TLR7, with TLR1, TLR6, TLR8, TLR10, TLR5, and TLR2 also upregulated. TLR3, TLR4, and TLR 9 were not differentially expressed. TLR7 is thought to be exclusively expressed by plasmacytoid dendritic cells, which secrete type I interferons in response to viral infection [23].

### Genes involved in lipid and lipoprotein metabolism are differentially expressed in KD arteritis

Many of the differentially expressed genes have functions in both lipid metabolism and immune response. Genes commonly associated with promotion of atherogenesis, such as LDLR and PLA2G2A, were downregulated. A notable feature was the downregulation of surfactant associated genes SFTPA1, SFTPA2, SFTPB, SFTPC, SFTPD, SFTA2, and SFTA3. It is possible that the marked downregulation in surfactant expression impairs the modulation of inflammatory responses in coronary artery smooth muscle cells in KD arteritis [24, 25].

### Cytokine and growth factor pathways that were not differentially expressed

Tumor necrosis factor receptor 1 and 2 signaling pathways were not differentially expressed (Fig. 3c). Transforming growth factor β signaling was also not differentially expressed. Interestingly, none of the following genes were differentially expressed in this dataset: matrix metalloproteinases (with the exception of MMP27, which just met significance criteria), vascular endothelial growth factors-A, B, or C, platelet derived growth factors-A,B,C, or D, vascular cell adhesion molecule 1, or fibroblast growth factor 2. The interleukin-1 signaling pathway was not differentially expressed. One IL-1 family member, IL-18, was upregulated. However, regulatory IL-1 family member IL1RN (IL1RA) was also upregulated, and IL1RL1 (IL33R, ST2) was markedly downregulated.

### RNA sequencing results are validated by real-time reverse transcriptase PCR assays

PCR assays confirmed significant upregulation of CD74, CD69, IL18, HLA-F, NLRC5, and CD226, genes involved in antigen presentation and dendritic cell function, in KD CA tissues (Additional file 1: Table S4). Genes that we previously identified as upregulated by real-time PCR on KD and control CA, including CD84, PIM2, POSTN, IL10RA, IL2RA, IQGAP2, and ITGA4 [26–28] were also identified as significantly upregulated by this transcriptome analysis, providing additional validation of our results.

### Differentially expressed genes encoding secreted proteins are candidate biomarkers of KD subacute/chronic arteritis

Extracellular genes encoding secreted proteins accounted for 155 of the differentially expressed genes (Additional file 1: Table S5), 93 were upregulated, and 13 were immunoglobulin genes. The other 80 proteins are candidate diagnostic/prognostic biomarkers of KD arteritis; such biomarkers are urgently needed for diagnosis and monitoring of KD patients. We have previously reported one of the upregulated proteins, periostin, as a potential diagnostic biomarker of KD [28].

### No known viral, bacterial, or fungal sequences were associated with KD

**Fig. 4:**
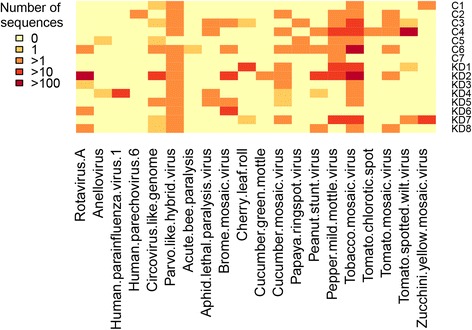
Heatmap showing viral sequences identified in KD and control coronary artery samples; none were KD-associated by Metastats analyses

Human and environmental viral sequences were identified in KD and control coronary artery samples, but none were associated with KD samples (Fig. 4). We identified the recently described parvovirus-circovirus hybrid virus as a contaminant in both KD and control datasets; this virus has previously been shown to contaminate certain nucleic acid isolation kits, and its initial report as a potential cause of human viral hepatitis highlights the importance of caution in performing pathogen discovery studies [29]. No specific bacterial or fungal sequences were associated with KD samples (data not shown).

## Discussion

Identification of upregulated immune pathways in CA target tissues significantly advances the understanding of KD pathogenesis, and in particular enhances knowledge of the molecular immunology of subacute/chronic coronary arteritis. Therapy aimed at reducing CA inflammation in KD patients has been largely empiric, because of a lack of information regarding the immunopathogenesis of disease in the target CA tissues.

Previous transcriptome studies have been performed only on peripheral blood of KD patients. Those studies revealed neutrophil activation prior to treatment with IGIV, consistent with elevated peripheral blood neutrophil counts during the acute febrile illness [19, 30], and consistent with the neutrophilic necrotizing arteritis of medium-sized muscular arteries such as the CA that occurs in the first two weeks after fever onset [4]. Identification of upregulated immune responses in the CA target tissues of KD provides previously unavailable information with potential therapeutic implications. A recent rigorous study demonstrated that fold changes in induced immune response proteins are particularly dominated by mRNA level changes, whereas expression of cytoskeletal, metabolic, ribosomal, and mitochondrial proteins are primarily controlled by translation and degradation rates [31], making it likely that the transcriptional upregulation identified in this study is accompanied by translational upregulation.

We found that T lymphocyte activation, antigen presentation and dendritic cell function, immunoglobulin production, and type I interferon response are the most significantly upregulated molecular pathways and processes in KD subacute/chronic arteritis. These are compatible with the gene polymorphisms resulting in a decrease in negative regulation of T lymphocyte responses that have been associated with the development of KD and CA abnormalities [32, 33]. It is also of interest that several type I interferon-induced proteins such as CXCL9 and CXCL10 are potential biomarkers of KD [34, 35].

Our study did not identify differential expression of the tumor necrosis factor α pathway, the transforming growth factor β pathway, matrix metalloproteinases-2,-3, −9 and-12, vascular endothelial growth factors, or platelet derived growth factors, which have been postulated previously as important players in the pathogenesis of KD arteritis by us and others [36–39]. These findings do not exclude a role for the previously proposed pathways and molecules in KD vasculitis pathogenesis, in particular because gene expression does not always correlate with protein expression. However, changes in mRNA abundance of immune response genes play the dominant role in dynamic changes in protein levels, whereas the proteome of proteins performing basic cellular functions are predominately regulated at the level of protein translation or degradation [31]. Therefore, the immune response genes found to be upregulated in our study may play the most prominent role in KD subacute/chronic vasculitis.

IGIV non-responders are a high-risk group of patients who are often administered additional immunomodulatory therapies with the goal of improving CA outcomes. However, identifying optimal therapies for such patients has been difficult, because the activated immune pathways in KD CA have been unknown. Because severe CA outcomes can occur even in IGIV-treated patients, particularly young infants, initial studies of combination primary therapies consisting of IGIV with a second immunomodulatory agent have been performed. A single dose of methylprednisolone with IGIV for primary therapy of KD did not improve outcomes [40], while in the more recent RAISE study, IGIV with a 3–4 week course of prednisolone for primary therapy of high-risk Japanese children did improve outcomes [41]. It seems plausible that a 2–4 week corticosteroid therapy course would be more effective in modulating the prominent T lymphocyte responses demonstrated in KD CA tissues in our study than would a single large dose of methylprednisolone. The use of a tumor necrosis factor-α inhibitor (infliximab) in combination with IGIV for primary therapy did not result in significantly improved outcomes [39]. The lack of differential expression of the tumor necrosis α pathway in the present study suggests that this cytokine may not be an optimal therapeutic target for subacute/chronic arteritis. Chronic type I interferon responses can be involved in the pathogenesis of persistent viral infections and autoimmune diseases [42, 43], and therapies to ameliorate this response are under study [44]. Because 3-hydroxy-3-methylglutaryl coenzyme A reductase inhibitors appear to have modulatory effects on cytotoxic T lymphocyte responses, therapeutic trials of these drugs in KD arteritis appear warranted [45].

Bioinformatics analysis did not reveal any known viral, bacterial, or fungal sequences associated with KD coronary artery tissues. There are many potential explanations for the lack of identification of a causative infectious agent in these tissues. The agent could be in a quantity too low to detect, or could have been eradicated by the immune response earlier in the disease course, with subacute/chronic arteritis the result of an inability to resolve the initial inflammatory response. Another possibility is a “new” agent not present in established reference databases, making it difficult to identify. Interestingly, our study revealed many upregulated type I interferon-induced genes in KD CA, which supports our prior hypothesis of a presently unidentified virus as the causative agent based on ultrastructural, immunofluorescence, and RNA evidence [2]. We are presently analyzing KD-associated sequences that remain unassigned in attempts to identify a putative “new” virus, using novel bioinformatics approaches, such as assembly without a reference genome [46].

Our study has strengths and limitations. The number of CA specimens available for study was limited by many factors. These factors include the fact that CA tissues are not available for research in the living patient, reporting of KD deaths is not required, autopsy is sometimes not obtained in fatal cases or is delayed so that RNA quality is inadequate for molecular studies, and heart tissues following transplantation are often not optimally preserved for molecular studies. Additional differentially expressed genes might have been identified if more CA tissues were available for sequencing. Because necrotizing arteritis is observed in the first 2 weeks after fever onset, when fatalities or surgical interventions are rare, our study would not reveal the transcriptome of necrotizing arteritis [4]. However, subacute/chronic arteritis begins in the first two weeks after fever onset and can persist for months to years, leading to significant CA damage [4]. Understanding its pathogenesis is particularly important for IGIV non-responders and for children with persistent coronary artery abnormalities, who may have chronic arteritis. KD patients in our study died or underwent cardiac transplant at various intervals since KD onset, at various ages, and had received a variety of therapies. However, gene expression did not cluster by these factors (Fig. 2b and c). Tissue samples were from Caucasian, Hispanic, and Black children but not Asian children, and from both genders, which could have affected gene expression results. As sequencing technology and bioinformatics methods improve, additional studies on larger numbers of KD CA samples, especially from Japan, would be informative to confirm and extend the present findings. Over several decades, we have developed what is likely the largest KD tissue bank outside of Japan, enabling us to identify the transcriptome of subacute/chronic KD arteritis in this study. A complete understanding of KD pathogenesis will require integration of genomic, transcriptome, and proteome studies, and identification of the etiologic agent(s).

## Conclusions

In summary, the immune transcriptome of KD arteritis in the patients studied here reveals a marked activation of cytotoxic and helper T lymphocytes and dendritic cells, with upregulation of type I interferon responses. These results support a putative viral etiology of KD, and provide preliminary information on the immunopathogenesis of KD coronary arteritis that can inform selection of new immunomodulatory therapies for clinical trials in high-risk patients with this potentially fatal arteritis of childhood.

Supporting data can be accessed at NCBI GEO GSE64486.

## Additional file

Additional file 1:
**Supplemental Methods.** **Table S1.** RNA sequencing metrics. **Table S2.** All differentially expressed genes (8 KD cases vs 7 controls). **Table S3.** Dysregulated molecular pathways (8 KD cases vs 7 controls). **Table S4.** Differential expression of genes involved in antigen presentation and dendritic cell function in KD compared to control coronary arteries by real-time reverse transcriptase PCR assays. **Table S5.** Extracellular genes dysregulated in 8 KD coronary arteries compared with 7 childhood control coronary arteries. Figure S1. Principal components analysis of all genes in 8 KD (4 treated and 4 untreated) and 7 childhood control coronary artery tissues demonstrates that gene expression of untreated (red dots) and treated (black dots) KD patients are not distinguishable. (PDF 382 kb)

## Abbreviations

KD: Kawasaki disease
CA: Coronary artery
