# Supplementary material for: The transcriptional profile of coronary arteritis in Kawasaki disease
Source: BMC Genomics. 2015 Dec 18;16:1076. doi: 10.1186/s12864-015-2323-5 (PMC4683744; doi:10.1186/s12864-015-2323-5)
Supplement: Additional file 1: — Supplemental Methods. Table S1. RNA sequencing metrics. Table S2. All differentially expressed genes (8 KD cases vs 7 controls). Table S3. Dysregulated molecular pathways (8 KD cases vs 7 controls). Table S4. Differential expression of genes involved in antigen presentation and dendritic cell function in KD compared to control coronary arteries by real-time reverse transcriptase PCR assays. Table S5. Extracellular genes dysregulated in 8 KD coronary arteries compared with 7 childhood control coronary arteries. Figure S1. Principal components analysis of all genes in 8 KD (4 treated and 4 untreated) and 7 childhood control coronary artery tissues demonstrates that gene expression of untreated (red dots) and treated (black dots) KD patients are not distinguishable. (PDF 382 kb) [file 12864_2015_2323_MOESM1_ESM.pdf]

## Supplemental Methods

**Patients and controls.** Coronary arteries from KD patients were individually embedded at autopsy/transplant, while control epicardial coronary arteries were microdissected from myocardial tissue blocks and re-embedded prior to sectioning. Tissues from patient KD4 became available after publication of our pathologic study; this child died suddenly 7 months after an illness consistent with incomplete KD. Coronary artery pathology in this patient revealed subacute/chronic inflammation with luminal myofibroblastic proliferation that totally obliterated the coronary artery lumen and resulted in myocardial infarction.

**RNA isolation and quality control analyses.** RNA was quantitated on a Nanodrop spectrophotometer (Thermo Scientific, Wilmington, DE). Samples were considered to be of good quality if the real-time PCR C(t) values for HGDC were at least 3 C(t) higher than for RPL13A (i.e., at least 8 fold more RNA than DNA was present in the sample), and the RPL13A C(t) was less than 32.

**High-throughput RNA sequencing.** Libraries were prepared using the Illumina Tru Seq RNA smaple prep kit, omitting RNA fragmentation because FFPE RNA was already fragmented.

**Pathways analysis.** Because the fragmented nature of the FFPE RNA could have made isoform analyses unreliable, we did not perform these analyses. iReport™ uses a right tailed Fisher's Exact test to determine the significance value (*p*-value) associated with the Biological Processes and Pathways in the Ingenuity Knowledge Base. The *p*-value is calculated by considering the number of differentially expressed genes in the dataset that participate in a specific annotation (Biological Process or Pathway), the number of genes in the Ingenuity Knowledge Base that participate in that specific annotation, the number of differentially

expressed genes that participate in any annotation, and the number of genes in the Ingenuity Knowledge Base.

**Real-time PCR.** Real-time PCR assays using primers for CD74, HLA-F, NLRC5, IL18, CD226, and CD69 were performed on KD and control coronary artery FFPE tissue RNA using the RT2 Pre-AMP cDNA synthesis kit (Qiagen, Valencia, CA). Reactions using primers for the housekeeping gene HPRT1 (Qiagen) were performed as an internal control, and primers for human genomic DNA as a quality control measure. For differential expression analysis, we used the comparative $C_T$ method[8], where $C_T$ is defined as the PCR cycle at which the fluorescent signal of the reporter dye crosses an arbitrarily placed threshold. The difference in expression levels of individual genes was determined by comparing $\Delta C_T$ values between the KD and control groups.

Supplemental Table 1. RNA sequencing metrics

| ID | qc passed reads | % mapped | % duplication | % UTR bases | % coding bases | % intergenic bases | % intronic bases | % ribosomal bases | % mRNA bases |
|---|---|---|---|---|---|---|---|---|---|
| **C1** | 79,849,136 | 86.38% | 17.4% | 20.6% | 10.9% | 6.0% | 59.6% | 3.0% | 31.5% |
| **C2** | 102,561,611 | 79.16% | 22.8% | 20.0% | 8.6% | 9.2% | 59.2% | 3.0% | 28.6% |
| **C3** | 113,905,139 | 74.02% | 41.2% | 14.4% | 7.2% | 17.2% | 59.8% | 1.4% | 21.6% |
| **C4** | 112,894,443 | 79.82% | 48.5% | 17.5% | 10.8% | 8.2% | 62.5% | 1.0% | 28.3% |
| **C5** | 84,373,360 | 86.20% | 22.4% | 18.7% | 14.2% | 5.3% | 57.0% | 4.7% | 33.0% |
| **C6** | 84,365,909 | 75.78% | 28.5% | 17.6% | 9.0% | 12.3% | 59.7% | 1.4% | 26.6% |
| **C7** | 109,410,697 | 90.82% | 29.1% | 20.7% | 15.1% | 19.6% | 38.2% | 6.5% | 35.8% |
| **KD1** | 84,779,783 | 50.20% | 65.0% | 9.3% | 2.7% | 57.7% | 30.2% | 0.1% | 12.0% |
| **KD2** | 122,931,914 | 53.22% | 58.4% | 13.1% | 5.9% | 30.0% | 50.9% | 0.1% | 19.0% |
| **KD3** | 81,854,092 | 87.77% | 14.0% | 15.2% | 10.3% | 8.6% | 62.5% | 3.4% | 25.5% |
| **KD4** | 40,419,542 | 49.95% | 38.8% | 36.6% | 8.4% | 7.8% | 47.1% | 0.0% | 45.1% |
| **KD5** | 92,878,797 | 85.79% | 19.8% | 14.9% | 6.7% | 6.4% | 71.0% | 0.9% | 21.6% |
| **KD6** | 99,048,879 | 84.68% | 21.4% | 20.5% | 9.9% | 6.0% | 62.7% | 0.9% | 30.4% |
| **KD7** | 94,342,947 | 60.87% | 43.1% | 15.1% | 6.9% | 11.2% | 66.5% | 0.3% | 22.0% |
| **KD8** | 119,207,003 | 84.02% | 25.9% | 22.3% | 11.2% | 7.1% | 52.7% | 6.7% | 33.5% |

**Supplemental Table 2. All differentially expressed genes (8 KD cases vs 7 controls)**

| Symbol: Name | Fold Change | Molecular Function | Location |
|---|---|---|---|
| CXCL9: chemokine (C-X-C motif) ligand 9 | 48.042 | cytokine | Extracellular Space |
| IGHG1: immunoglobulin heavy constant gamma 1 (G1m marker) | 44.986 | other | Extracellular Space |
| OLR1: oxidized low density lipoprotein (lectin-like) receptor 1 | 42.163 | transmembrane receptor | Plasma Membrane |
| IGHM: immunoglobulin heavy constant mu | 34.153 | transmembrane receptor | Plasma Membrane |
| IGJ: immunoglobulin J polypeptide, linker protein for immunoglobulin alpha and mu polypeptic | 31.305 | other | Extracellular Space |
| CXCL13: chemokine (C-X-C motif) ligand 13 | 21.829 | cytokine | Extracellular Space |
| FCRL5: Fc receptor-like 5 | 21.053 | other | Plasma Membrane |
| ADAMDEC1: ADAM-like, decysin 1 | 20.541 | peptidase | Extracellular Space |
| SFRP4: secreted frizzled-related protein 4 | 19.065 | transmembrane receptor | Plasma Membrane |
| BCL11B: B-cell CLL/lymphoma 11B (zinc finger protein) | 17.604 | other | Nucleus |
| CD3E: CD3e molecule, epsilon (CD3-TCR complex) | 17.498 | transmembrane receptor | Plasma Membrane |
| IGHG3: immunoglobulin heavy constant gamma 3 (G3m marker) | 16.976 | other | Extracellular Space |
| RGS1: regulator of G-protein signaling 1 | 16.976 | other | Plasma Membrane |
| HLA-DPA1: major histocompatibility complex, class II, DP alpha 1 | 16.753 | other | Cytoplasm |
| FCGR3A/FCGR3B: Fc fragment of IgG, low affinity IIIa, receptor (CD16a) | 16.736 | transmembrane receptor | Plasma Membrane |
| FYB: FYN binding protein | 16.498 | other | Nucleus |
| SFRP2: secreted frizzled-related protein 2 | 16.159 | transmembrane receptor | Plasma Membrane |
| IGKV3-20: immunoglobulin kappa variable 3-20 | 16.027 | other | Extracellular Space |
| CD3G: CD3g molecule, gamma (CD3-TCR complex) | 15.831 | transmembrane receptor | Plasma Membrane |
| THEMIS: thymocyte selection associated | 15.563 | other | Cytoplasm |
| HLA-DQA1: major histocompatibility complex, class II, DQ alpha 1 | 15.359 | transmembrane receptor | Plasma Membrane |
| CXCR4: chemokine (C-X-C motif) receptor 4 | 14.728 | G-protein coupled recepto | Plasma Membrane |
| GBP5: guanylate binding protein 5 | 14.67 | enzyme | Plasma Membrane |
| MS4A1: membrane-spanning 4-domains, subfamily A, member 1 | 14.228 | other | Plasma Membrane |
| SELL: selectin L | 14.025 | transmembrane receptor | Plasma Membrane |
| IGHG2: immunoglobulin heavy constant gamma 2 (G2m marker) | 13.666 | other | Plasma Membrane |
| FAP: fibroblast activation protein, alpha | 13.533 | peptidase | Cytoplasm |
| IKZF3: IKAROS family zinc finger 3 (Aiolos) | 13.166 | transcription regulator | Nucleus |
| CD2: CD2 molecule | 12.902 | transmembrane receptor | Plasma Membrane |
| SLAMF7: SLAM family member 7 | 12.834 | other | Plasma Membrane |
| IGHA1: immunoglobulin heavy constant alpha 1 | 12.818 | other | Extracellular Space |
| IGHG4: immunoglobulin heavy constant gamma 4 (G4m marker) | 12.528 | other | Extracellular Space |
| IGLC3: immunoglobulin lambda constant 3 (Kern-Oz+ marker) | 11.926 | other | Extracellular Space |
| LEF1: lymphoid enhancer-binding factor 1 | 11.912 | transcription regulator | Nucleus |
| TRAC: T cell receptor alpha constant | 11.498 | other | Plasma Membrane |
| IGKV1-5: immunoglobulin kappa variable 1-5 | 11.474 | other | Extracellular Space |

| | | | |
|---|---|---|---|
| COMP: cartilage oligomeric matrix protein | 11.372 | other | Extracellular Space |
| CD8B: CD8b molecule | 11.371 | other | Plasma Membrane |
| TLR7: toll-like receptor 7 | 11.123 | transmembrane receptor | Plasma Membrane |
| CD84: CD84 molecule | 10.954 | other | Plasma Membrane |
| ITGA4: integrin, alpha 4 (antigen CD49D, alpha 4 subunit of VLA-4 receptor) | 10.925 | transmembrane receptor | Plasma Membrane |
| ITK: IL2-inducible T-cell kinase | 10.69 | kinase | Cytoplasm |
| CCL18: chemokine (C-C motif) ligand 18 (pulmonary and activation-regulated) | 10.596 | cytokine | Extracellular Space |
| CYBB: cytochrome b-245, beta polypeptide | 10.52 | enzyme | Cytoplasm |
| SCML4: sex comb on midleg-like 4 (Drosophila) | 10.465 | other | Unknown |
| ACAN: aggrecan | 10.357 | other | Extracellular Space |
| CCR2: chemokine (C-C motif) receptor 2 | 10.336 | G-protein coupled recepto | Plasma Membrane |
| IGLC2: immunoglobulin lambda constant 2 (Kern-Oz- marker) | 10.326 | other | Extracellular Space |
| CD69: CD69 molecule | 10.313 | transmembrane receptor | Plasma Membrane |
| CYTIP: cytohesin 1 interacting protein | 10.25 | other | Cytoplasm |
| ANKRD22: ankyrin repeat domain 22 | 10.182 | transcription regulator | Nucleus |
| SH2D1A: SH2 domain containing 1A | 10.135 | other | Cytoplasm |
| MS4A6A: membrane-spanning 4-domains, subfamily A, member 6A | 10.082 | other | Unknown |
| LINC00861: long intergenic non-protein coding RNA 861 | 10.055 | other | Unknown |
| IKZF1: IKAROS family zinc finger 1 (Ikaros) | 10.035 | transcription regulator | Nucleus |
| HLA-DQB1: major histocompatibility complex, class II, DQ beta 1 | 10.018 | other | Plasma Membrane |
| AMICA1: adhesion molecule, interacts with CXADR antigen 1 | 9.923 | other | Plasma Membrane |
| FCGR2C: Fc fragment of IgG, low affinity IIc, receptor for (CD32) (gene/pseudogene) | 9.895 | transmembrane receptor | Plasma Membrane |
| BTLA: B and T lymphocyte associated | 9.879 | other | Plasma Membrane |
| HLA-DOA: major histocompatibility complex, class II, DO alpha | 9.821 | transmembrane receptor | Plasma Membrane |
| HLA-DRA: major histocompatibility complex, class II, DR alpha | 9.491 | transmembrane receptor | Plasma Membrane |
| IL7R: interleukin 7 receptor | 9.424 | transmembrane receptor | Plasma Membrane |
| LOC101929010: uncharacterized LOC101929010 | 9.318 | other | Unknown |
| TIMD4: T-cell immunoglobulin and mucin domain containing 4 | 9.305 | other | Plasma Membrane |
| ADAM28: ADAM metallopeptidase domain 28 | 9.283 | peptidase | Plasma Membrane |
| PRND: prion protein 2 (dublet) | 9.027 | other | Plasma Membrane |
| HLA-DMB: major histocompatibility complex, class II, DM beta | 8.917 | other | Cytoplasm |
| NELL2: NEL-like 2 (chicken) | 8.779 | other | Extracellular Space |
| IGHV1-69: immunoglobulin heavy variable 1-69 | 8.749 | other | Unknown |
| P2RY10: purinergic receptor P2Y, G-protein coupled, 10 | 8.704 | G-protein coupled recepto | Plasma Membrane |
| ICOS: inducible T-cell co-stimulator | 8.655 | transmembrane receptor | Plasma Membrane |
| SLAMF6: SLAM family member 6 | 8.616 | transmembrane receptor | Plasma Membrane |
| CERKL: ceramide kinase-like | 8.603 | other | Unknown |
| IL2RG: interleukin 2 receptor, gamma | 8.537 | transmembrane receptor | Plasma Membrane |

| | | | |
|---|---|---|---|
| ARL4C: ADP-ribosylation factor-like 4C | 8.525 | enzyme | Nucleus |
| PTPRC: protein tyrosine phosphatase, receptor type, C | 8.516 | phosphatase | Plasma Membrane |
| FCRL3: Fc receptor-like 3 | 8.493 | other | Unknown |
| LYZ: lysozyme | 8.366 | enzyme | Extracellular Space |
| CD3D: CD3d molecule, delta (CD3-TCR complex) | 8.251 | transmembrane receptor | Plasma Membrane |
| LY9: lymphocyte antigen 9 | 8.249 | other | Plasma Membrane |
| TRAF3IP3: TRAF3 interacting protein 3 | 8.238 | other | Unknown |
| MXRA5: matrix-remodelling associated 5 | 8.125 | other | Extracellular Space |
| CXCL14: chemokine (C-X-C motif) ligand 14 | 8.122 | cytokine | Extracellular Space |
| DAPP1: dual adaptor of phosphotyrosine and 3-phosphoinositides | 8.121 | other | Cytoplasm |
| CD8A: CD8a molecule | 8.066 | other | Plasma Membrane |
| SAMD3: sterile alpha motif domain containing 3 | 7.919 | other | Unknown |
| HLA-DPB1: major histocompatibility complex, class II, DP beta 1 | 7.856 | transmembrane receptor | Plasma Membrane |
| LINC01094: long intergenic non-protein coding RNA 1094 | 7.759 | other | Unknown |
| RHOH: ras homolog family member H | 7.753 | enzyme | Plasma Membrane |
| LCP2: lymphocyte cytosolic protein 2 (SH2 domain containing leukocyte protein of 76kDa) | 7.739 | other | Cytoplasm |
| CD74: CD74 molecule, major histocompatibility complex, class II invariant chain | 7.738 | transmembrane receptor | Plasma Membrane |
| SORL1: sortilin-related receptor, L(DLR class) A repeats containing | 7.655 | transporter | Cytoplasm |
| EMB: embigin | 7.651 | other | Plasma Membrane |
| KCNA3: potassium voltage-gated channel, shaker-related subfamily, member 3 | 7.638 | ion channel | Plasma Membrane |
| CD96: CD96 molecule | 7.62 | other | Plasma Membrane |
| TXK: TXK tyrosine kinase | 7.587 | kinase | Cytoplasm |
| GAPT: GRB2-binding adaptor protein, transmembrane | 7.481 | other | Unknown |
| COL11A1: collagen, type XI, alpha 1 | 7.467 | other | Extracellular Space |
| STAP1: signal transducing adaptor family member 1 | 7.409 | other | Cytoplasm |
| TFEC: transcription factor EC | 7.37 | transcription regulator | Nucleus |
| IGKC: immunoglobulin kappa constant | 7.269 | other | Extracellular Space |
| IL2RA: interleukin 2 receptor, alpha | 7.251 | transmembrane receptor | Plasma Membrane |
| TESPA1: thymocyte expressed, positive selection associated 1 | 7.205 | other | Cytoplasm |
| IFNG-AS1: IFNG antisense RNA 1 | 7.191 | other | Unknown |
| MNDA: myeloid cell nuclear differentiation antigen | 7.126 | other | Nucleus |
| C5orf46: chromosome 5 open reading frame 46 | 7.117 | other | Cytoplasm |
| CD6: CD6 molecule | 7.117 | transmembrane receptor | Plasma Membrane |
| GPR174: G protein-coupled receptor 174 | 7.082 | G-protein coupled recepto | Plasma Membrane |
| FPR3: formyl peptide receptor 3 | 7.071 | other | Plasma Membrane |
| IGHV3-21: immunoglobulin heavy variable 3-21 | 7.029 | other | Unknown |
| LAX1: lymphocyte transmembrane adaptor 1 | 6.995 | other | Cytoplasm |
| RNASE6: ribonuclease, RNase A family, k6 | 6.945 | enzyme | Extracellular Space |

| | | | |
|---|---|---|---|
| LILRB4: leukocyte immunoglobulin-like receptor, subfamily B (with TM and ITIM domains), me | 6.789 | other | Plasma Membrane |
| IL2RB: interleukin 2 receptor, beta | 6.737 | transmembrane receptor | Plasma Membrane |
| LOC101060038: uncharacterized LOC101060038 | 6.68 | other | Unknown |
| CD53: CD53 molecule | 6.634 | other | Plasma Membrane |
| PNRC2: proline-rich nuclear receptor coactivator 2 | 6.591 | other | Nucleus |
| POSTN: periostin, osteoblast specific factor | 6.53 | other | Extracellular Space |
| CCL5: chemokine (C-C motif) ligand 5 | 6.529 | cytokine | Extracellular Space |
| CCR5: chemokine (C-C motif) receptor 5 (gene/pseudogene) | 6.501 | G-protein coupled recepto | Plasma Membrane |
| FAM177B: family with sequence similarity 177, member B | 6.496 | other | Unknown |
| CAMK4: calcium/calmodulin-dependent protein kinase IV | 6.485 | kinase | Nucleus |
| PTPN22: protein tyrosine phosphatase, non-receptor type 22 (lymphoid) | 6.481 | phosphatase | Cytoplasm |
| CTSS: cathepsin S | 6.454 | peptidase | Cytoplasm |
| HOXC6: homeobox C6 | 6.454 | transcription regulator | Nucleus |
| TMEM156: transmembrane protein 156 | 6.433 | other | Unknown |
| IGKV4-1: immunoglobulin kappa variable 4-1 | 6.432 | other | Extracellular Space |
| LOC100996286: uncharacterized LOC100996286 | 6.418 | other | Unknown |
| PRSS35: protease, serine, 35 | 6.407 | peptidase | Extracellular Space |
| TMEM52B: transmembrane protein 52B | 6.401 | other | Cytoplasm |
| CRTAM: cytotoxic and regulatory T cell molecule | 6.381 | other | Plasma Membrane |
| CD86: CD86 molecule | 6.379 | transmembrane receptor | Plasma Membrane |
| P2RY13: purinergic receptor P2Y, G-protein coupled, 13 | 6.356 | G-protein coupled recepto | Plasma Membrane |
| LINC00426: long intergenic non-protein coding RNA 426 | 6.337 | other | Unknown |
| PRKCB: protein kinase C, beta | 6.333 | kinase | Cytoplasm |
| IGLV3-21: immunoglobulin lambda variable 3-21 | 6.293 | other | Unknown |
| MS4A4E: membrane-spanning 4-domains, subfamily A, member 4E | 6.267 | other | Unknown |
| POU2AF1: POU class 2 associating factor 1 | 6.265 | transcription regulator | Nucleus |
| LAPTM5: lysosomal protein transmembrane 5 | 6.262 | other | Plasma Membrane |
| CX3CR1: chemokine (C-X3-C motif) receptor 1 | 6.203 | G-protein coupled recepto | Plasma Membrane |
| SCG2: secretogranin II | 6.189 | cytokine | Extracellular Space |
| EGFL6: EGF-like-domain, multiple 6 | 6.184 | other | Extracellular Space |
| SNX10: sorting nexin 10 | 6.175 | transporter | Cytoplasm |
| TAGAP: T-cell activation RhoGTPase activating protein | 6.165 | other | Cytoplasm |
| PLEK: pleckstrin | 6.104 | other | Cytoplasm |
| LCK: lymphocyte-specific protein tyrosine kinase | 6.075 | kinase | Cytoplasm |
| SP140: SP140 nuclear body protein | 6.057 | transcription regulator | Nucleus |
| RGS18: regulator of G-protein signaling 18 | 6.033 | other | Cytoplasm |
| DOCK8: dedicator of cytokinesis 8 | 6.02 | other | Cytoplasm |
| AOAH: acyloxyacyl hydrolase (neutrophil) | 5.987 | enzyme | Extracellular Space |

| | | | |
|---|---|---|---|
| ANKRD36BP2: ankyrin repeat domain 36B pseudogene 2 | 5.895 | other | Unknown |
| LOC101927156: uncharacterized LOC101927156 | 5.894 | other | Unknown |
| CXCR2P1: chemokine (C-X-C motif) receptor 2 pseudogene 1 | 5.89 | other | Unknown |
| PLA2G7: phospholipase A2, group VII (platelet-activating factor acetylhydrolase, plasma) | 5.878 | enzyme | Extracellular Space |
| DOCK10: dedicator of cytokinesis 10 | 5.874 | other | Cytoplasm |
| SULF1: sulfatase 1 | 5.823 | enzyme | Cytoplasm |
| FCGR2A: Fc fragment of IgG, low affinity IIa, receptor (CD32) | 5.821 | transmembrane receptor | Plasma Membrane |
| GRAP2: GRB2-related adaptor protein 2 | 5.809 | other | Cytoplasm |
| MSR1: macrophage scavenger receptor 1 | 5.762 | transmembrane receptor | Plasma Membrane |
| PLAC8: placenta-specific 8 | 5.751 | other | Nucleus |
| CD48: CD48 molecule | 5.716 | other | Plasma Membrane |
| CR2: complement component (3d/Epstein Barr virus) receptor 2 | 5.709 | transmembrane receptor | Plasma Membrane |
| CXorf21: chromosome X open reading frame 21 | 5.69 | enzyme | Unknown |
| ITGAL: integrin, alpha L (antigen CD11A (p180), lymphocyte function-associated antigen 1; a | 5.68 | transmembrane receptor | Plasma Membrane |
| CLEC5A: C-type lectin domain family 5, member A | 5.653 | other | Plasma Membrane |
| IL21R: interleukin 21 receptor | 5.646 | transmembrane receptor | Plasma Membrane |
| ITGB2-AS1: ITGB2 antisense RNA 1 | 5.645 | other | Unknown |
| ZBP1: Z-DNA binding protein 1 | 5.643 | other | Cytoplasm |
| SUSD3: sushi domain containing 3 | 5.627 | other | Unknown |
| SULT1E1: sulfotransferase family 1E, estrogen-preferring, member 1 | 5.626 | enzyme | Cytoplasm |
| FCRL2: Fc receptor-like 2 | 5.588 | other | Unknown |
| GPR141: G protein-coupled receptor 141 | 5.55 | G-protein coupled recepto | Plasma Membrane |
| TRABD2A: TraB domain containing 2A | 5.549 | peptidase | Plasma Membrane |
| MYB: v-myb avian myeloblastosis viral oncogene homolog | 5.549 | transcription regulator | Nucleus |
| CD247: CD247 molecule | 5.533 | transmembrane receptor | Plasma Membrane |
| CD52: CD52 molecule | 5.484 | other | Plasma Membrane |
| TRAT1: T cell receptor associated transmembrane adaptor 1 | 5.478 | kinase | Plasma Membrane |
| AIF1: allograft inflammatory factor 1 | 5.463 | other | Nucleus |
| IPCEF1: interaction protein for cytohesin exchange factors 1 | 5.455 | enzyme | Cytoplasm |
| RNASET2: ribonuclease T2 | 5.444 | enzyme | Cytoplasm |
| IGLV3-25: immunoglobulin lambda variable 3-25 | 5.435 | other | Unknown |
| CA12: carbonic anhydrase XII | 5.406 | enzyme | Plasma Membrane |
| PRKCQ-AS1: PRKCQ antisense RNA 1 | 5.388 | other | Unknown |
| XCL1: chemokine (C motif) ligand 1 | 5.357 | cytokine | Extracellular Space |
| CD4: CD4 molecule | 5.349 | transmembrane receptor | Plasma Membrane |
| BTBD11: BTB (POZ) domain containing 11 | 5.333 | transcription regulator | Unknown |
| LCP1: lymphocyte cytosolic protein 1 (L-plastin) | 5.33 | other | Cytoplasm |
| MIAT: myocardial infarction associated transcript (non-protein coding) | 5.307 | other | Unknown |

| | | | |
|---|---|---|---|
| IGHV4-39: immunoglobulin heavy variable 4-39 | 5.294 | other | Unknown |
| ZBED2: zinc finger, BED-type containing 2 | 5.282 | other | Unknown |
| IGLV1-40: immunoglobulin lambda variable 1-40 | 5.268 | other | Unknown |
| FAM46C: family with sequence similarity 46, member C | 5.251 | other | Extracellular Space |
| TLR1: toll-like receptor 1 | 5.239 | transmembrane receptor | Plasma Membrane |
| PAX5: paired box 5 | 5.213 | transcription regulator | Nucleus |
| TLR6: toll-like receptor 6 | 5.168 | transmembrane receptor | Plasma Membrane |
| UBASH3A: ubiquitin associated and SH3 domain containing A | 5.13 | enzyme | Cytoplasm |
| CD226: CD226 molecule | 5.094 | other | Plasma Membrane |
| FNDC1: fibronectin type III domain containing 1 | 5.077 | other | Plasma Membrane |
| ARHGAP15: Rho GTPase activating protein 15 | 5.052 | other | Cytoplasm |
| IL10RA: interleukin 10 receptor, alpha | 5.021 | transmembrane receptor | Plasma Membrane |
| SKAP1: src kinase associated phosphoprotein 1 | 5.017 | kinase | Cytoplasm |
| IGHA2: immunoglobulin heavy constant alpha 2 (A2m marker) | 5.016 | other | Extracellular Space |
| SIGLEC10: sialic acid binding Ig-like lectin 10 | 5.01 | other | Plasma Membrane |
| LRMP: lymphoid-restricted membrane protein | 4.988 | other | Cytoplasm |
| GPR65: G protein-coupled receptor 65 | 4.986 | G-protein coupled recepto | Plasma Membrane |
| CLEC10A: C-type lectin domain family 10, member A | 4.985 | other | Plasma Membrane |
| OAS2: 2'-5'-oligoadenylate synthetase 2, 69/71kDa | 4.964 | enzyme | Cytoplasm |
| AQP9: aquaporin 9 | 4.939 | transporter | Plasma Membrane |
| TMEM200A: transmembrane protein 200A | 4.934 | other | Unknown |
| SPIB: Spi-B transcription factor (Spi-1/PU.1 related) | 4.933 | transcription regulator | Nucleus |
| HORMAD1: HORMA domain containing 1 | 4.93 | other | Nucleus |
| EPSTI1: epithelial stromal interaction 1 (breast) | 4.918 | other | Unknown |
| GVINP1: GTPase, very large interferon inducible pseudogene 1 | 4.898 | other | Unknown |
| ITGBL1: integrin, beta-like 1 (with EGF-like repeat domains) | 4.885 | other | Unknown |
| SIGLEC1: sialic acid binding Ig-like lectin 1, sialoadhesin | 4.875 | other | Plasma Membrane |
| LOC101928100: uncharacterized LOC101928100 | 4.867 | other | Unknown |
| CD28: CD28 molecule | 4.848 | transmembrane receptor | Plasma Membrane |
| DOCK2: dedicator of cytokinesis 2 | 4.848 | other | Cytoplasm |
| C5orf58: chromosome 5 open reading frame 58 | 4.841 | other | Unknown |
| BLNK: B-cell linker | 4.825 | other | Cytoplasm |
| MAP4K1: mitogen-activated protein kinase kinase kinase kinase 1 | 4.823 | kinase | Cytoplasm |
| RUNX3: runt-related transcription factor 3 | 4.819 | transcription regulator | Nucleus |
| TLR8: toll-like receptor 8 | 4.806 | transmembrane receptor | Plasma Membrane |
| IGHD: immunoglobulin heavy constant delta | 4.793 | other | Extracellular Space |
| ZNF683: zinc finger protein 683 | 4.77 | other | Unknown |
| IQGAP2: IQ motif containing GTPase activating protein 2 | 4.753 | other | Cytoplasm |

| | | | |
|---|---|---|---|
| MX2: myxovirus (influenza virus) resistance 2 (mouse) | 4.746 | enzyme | Nucleus |
| ARHGAP30: Rho GTPase activating protein 30 | 4.745 | other | Cytoplasm |
| TMC8: transmembrane channel-like 8 | 4.725 | other | Cytoplasm |
| BCL2A1: BCL2-related protein A1 | 4.724 | other | Cytoplasm |
| TNFSF8: tumor necrosis factor (ligand) superfamily, member 8 | 4.722 | cytokine | Plasma Membrane |
| SAMD9L: sterile alpha motif domain containing 9-like | 4.718 | other | Extracellular Space |
| FAM159A: family with sequence similarity 159, member A | 4.717 | other | Unknown |
| PIM2: pim-2 oncogene | 4.715 | kinase | Unknown |
| CECR1: cat eye syndrome chromosome region, candidate 1 | 4.698 | enzyme | Extracellular Space |
| CTLA4: cytotoxic T-lymphocyte-associated protein 4 | 4.681 | transmembrane receptor | Plasma Membrane |
| DENND2D: DENN/MADD domain containing 2D | 4.669 | other | Cytoplasm |
| KYNU: kynureninase | 4.668 | enzyme | Cytoplasm |
| FCN1: ficolin (collagen/fibrinogen domain containing) 1 | 4.657 | other | Extracellular Space |
| ARRDC5: arrestin domain containing 5 | 4.653 | other | Unknown |
| LTBP2: latent transforming growth factor beta binding protein 2 | 4.652 | other | Extracellular Space |
| LY75: lymphocyte antigen 75 | 4.641 | transmembrane receptor | Plasma Membrane |
| FCRL1: Fc receptor-like 1 | 4.626 | other | Unknown |
| RGS2: regulator of G-protein signaling 2, 24kDa | 4.612 | other | Nucleus |
| PIK3CG: phosphatidylinositol-4,5-bisphosphate 3-kinase, catalytic subunit gamma | 4.601 | kinase | Cytoplasm |
| IFI30: interferon, gamma-inducible protein 30 | 4.576 | enzyme | Cytoplasm |
| SAMD9: sterile alpha motif domain containing 9 | 4.575 | other | Cytoplasm |
| CD79A: CD79a molecule, immunoglobulin-associated alpha | 4.573 | transmembrane receptor | Plasma Membrane |
| PYHIN1: pyrin and HIN domain family, member 1 | 4.565 | other | Nucleus |
| GNLY: granulysin | 4.556 | other | Cytoplasm |
| EVI2B: ecotropic viral integration site 2B | 4.544 | other | Plasma Membrane |
| OLFML2B: olfactomedin-like 2B | 4.52 | other | Extracellular Space |
| ST8SIA4: ST8 alpha-N-acetyl-neuraminide alpha-2,8-sialyltransferase 4 | 4.512 | enzyme | Cytoplasm |
| CIITA: class II, major histocompatibility complex, transactivator | 4.499 | transcription regulator | Nucleus |
| KIAA0125: KIAA0125 | 4.487 | other | Unknown |
| PRDM1: PR domain containing 1, with ZNF domain | 4.475 | transcription regulator | Nucleus |
| CD7: CD7 molecule | 4.466 | other | Plasma Membrane |
| C14orf182: chromosome 14 open reading frame 182 | 4.464 | other | Unknown |
| DMP1: dentin matrix acidic phosphoprotein 1 | 4.449 | other | Extracellular Space |
| CENPE: centromere protein E, 312kDa | 4.441 | other | Nucleus |
| MS4A14: membrane-spanning 4-domains, subfamily A, member 14 | 4.434 | other | Unknown |
| SNX20: sorting nexin 20 | 4.415 | other | Unknown |
| GZMK: granzyme K (granzyme 3; tryptase II) | 4.411 | peptidase | Cytoplasm |
| NPL: N-acetylneuraminate pyruvate lyase (dihydrodipicolinate synthase) | 4.401 | enzyme | Unknown |

| | | | |
|---|---|---|---|
| LOC100132612: uncharacterized LOC100132612 | 4.383 | other | Unknown |
| CSF2RA: colony stimulating factor 2 receptor, alpha, low-affinity (granulocyte-macrophage) | 4.368 | transmembrane receptor | Plasma Membrane |
| DPEP2: dipeptidase 2 | 4.367 | peptidase | Plasma Membrane |
| PSD4: pleckstrin and Sec7 domain containing 4 | 4.365 | other | Cytoplasm |
| CLEC12A: C-type lectin domain family 12, member A | 4.363 | other | Plasma Membrane |
| LEF1-AS1: LEF1 antisense RNA 1 | 4.36 | other | Unknown |
| GGTA1P: glycoprotein, alpha-galactosyltransferase 1 pseudogene | 4.359 | other | Plasma Membrane |
| CCR4: chemokine (C-C motif) receptor 4 | 4.334 | G-protein coupled recepto | Plasma Membrane |
| ERAP2: endoplasmic reticulum aminopeptidase 2 | 4.324 | peptidase | Cytoplasm |
| TMEM130: transmembrane protein 130 | 4.322 | other | Unknown |
| AOAH-IT1: AOAH intronic transcript 1 (non-protein coding) | 4.314 | other | Unknown |
| TENM1: teneurin transmembrane protein 1 | 4.314 | peptidase | Plasma Membrane |
| NCF2: neutrophil cytosolic factor 2 | 4.307 | enzyme | Cytoplasm |
| ANKRD44-IT1: ANKRD44 intronic transcript 1 (non-protein coding) | 4.303 | other | Unknown |
| FAM26F: family with sequence similarity 26, member F | 4.297 | other | Unknown |
| GBP2: guanylate binding protein 2, interferon-inducible | 4.297 | enzyme | Cytoplasm |
| NCKAP1L: NCK-associated protein 1-like | 4.292 | other | Plasma Membrane |
| RAB39B: RAB39B, member RAS oncogene family | 4.28 | enzyme | Plasma Membrane |
| OAS1: 2'-5'-oligoadenylate synthetase 1, 40/46kDa | 4.274 | enzyme | Cytoplasm |
| IDO2: indoleamine 2,3-dioxygenase 2 | 4.273 | enzyme | Cytoplasm |
| KLHL6: kelch-like family member 6 | 4.269 | other | Unknown |
| KLRC4-KLRK1/KLRK1: killer cell lectin-like receptor subfamily K, member 1 | 4.269 | transmembrane receptor | Plasma Membrane |
| RASAL3: RAS protein activator like 3 | 4.269 | other | Cytoplasm |
| ARHGDIB: Rho GDP dissociation inhibitor (GDI) beta | 4.268 | other | Cytoplasm |
| FLT3: fms-related tyrosine kinase 3 | 4.259 | kinase | Plasma Membrane |
| CLEC7A: C-type lectin domain family 7, member A | 4.243 | transmembrane receptor | Plasma Membrane |
| SEMA4D: sema domain, immunoglobulin domain (Ig), transmembrane domain (TM) and shor | 4.229 | transmembrane receptor | Plasma Membrane |
| MX1: myxovirus (influenza virus) resistance 1, interferon-inducible protein p78 (mouse) | 4.228 | enzyme | Cytoplasm |
| SIRPG: signal-regulatory protein gamma | 4.226 | other | Plasma Membrane |
| CD300A: CD300a molecule | 4.216 | transmembrane receptor | Plasma Membrane |
| HCLS1: hematopoietic cell-specific Lyn substrate 1 | 4.199 | other | Nucleus |
| GPR114: G protein-coupled receptor 114 | 4.189 | G-protein coupled recepto | Plasma Membrane |
| BTN2A2: butyrophilin, subfamily 2, member A2 | 4.183 | other | Unknown |
| TNFSF13B: tumor necrosis factor (ligand) superfamily, member 13b | 4.171 | cytokine | Extracellular Space |
| IFI44L: interferon-induced protein 44-like | 4.166 | other | Unknown |
| BTN3A1: butyrophilin, subfamily 3, member A1 | 4.138 | other | Extracellular Space |
| RSAD2: radical S-adenosyl methionine domain containing 2 | 4.133 | enzyme | Cytoplasm |
| LOC100507616: uncharacterized LOC100507616 | 4.127 | other | Unknown |

| | | | |
|---|---|---|---|
| RGS10: regulator of G-protein signaling 10 | 4.118 | other | Cytoplasm |
| ZC3H12D: zinc finger CCCH-type containing 12D | 4.11 | other | Cytoplasm |
| EMR3: egf-like module containing, mucin-like, hormone receptor-like 3 | 4.106 | G-protein coupled recepto | Plasma Membrane |
| BIN2: bridging integrator 2 | 4.104 | other | Plasma Membrane |
| ERMN: ermin, ERM-like protein | 4.086 | other | Extracellular Space |
| TLR10: toll-like receptor 10 | 4.057 | transmembrane receptor | Plasma Membrane |
| NCF1: neutrophil cytosolic factor 1 | 4.054 | enzyme | Cytoplasm |
| FAIM3: Fas apoptotic inhibitory molecule 3 | 4.053 | other | Plasma Membrane |
| C16orf54: chromosome 16 open reading frame 54 | 4.033 | other | Extracellular Space |
| CD72: CD72 molecule | 4.032 | transmembrane receptor | Plasma Membrane |
| ASPN: asporin | 4.025 | other | Extracellular Space |
| SLFN12L: schlafen family member 12-like | 4.023 | enzyme | Unknown |
| CCDC88B: coiled-coil domain containing 88B | 4.021 | enzyme | Nucleus |
| STK17B: serine/threonine kinase 17b | 4.021 | kinase | Nucleus |
| TNFSF11: tumor necrosis factor (ligand) superfamily, member 11 | 4.008 | cytokine | Extracellular Space |
| FCER1G: Fc fragment of IgE, high affinity I, receptor for; gamma polypeptide | 4.004 | transmembrane receptor | Plasma Membrane |
| ITGAX: integrin, alpha X (complement component 3 receptor 4 subunit) | 4 | transmembrane receptor | Plasma Membrane |
| IKZF2: IKAROS family zinc finger 2 (Helios) | 3.992 | transcription regulator | Nucleus |
| NME8: NME/NM23 family member 8 | 3.985 | kinase | Cytoplasm |
| C15orf48: chromosome 15 open reading frame 48 | 3.97 | other | Nucleus |
| C1orf200: chromosome 1 open reading frame 200 | 3.955 | other | Unknown |
| CCDC64: coiled-coil domain containing 64 | 3.95 | other | Cytoplasm |
| FASLG: Fas ligand (TNF superfamily, member 6) | 3.949 | cytokine | Extracellular Space |
| SMAP2: small ArfGAP2 | 3.948 | other | Unknown |
| HLA-DMA: major histocompatibility complex, class II, DM alpha | 3.947 | other | Cytoplasm |
| CDH11: cadherin 11, type 2, OB-cadherin (osteoblast) | 3.929 | other | Plasma Membrane |
| LOC100130476: uncharacterized LOC100130476 | 3.922 | other | Unknown |
| KRT14: keratin 14 | 3.904 | other | Cytoplasm |
| SHOX2: short stature homeobox 2 | 3.904 | transcription regulator | Nucleus |
| CLEC4E: C-type lectin domain family 4, member E | 3.901 | other | Plasma Membrane |
| CD1C: CD1c molecule | 3.897 | other | Plasma Membrane |
| SELPLG: selectin P ligand | 3.895 | other | Plasma Membrane |
| KLRC1: killer cell lectin-like receptor subfamily C, member 1 | 3.894 | transmembrane receptor | Plasma Membrane |
| EVI2A: ecotropic viral integration site 2A | 3.883 | transmembrane receptor | Plasma Membrane |
| ADAM8: ADAM metallopeptidase domain 8 | 3.87 | peptidase | Plasma Membrane |
| ARHGAP9: Rho GTPase activating protein 9 | 3.867 | other | Cytoplasm |
| HLA-DRB5: major histocompatibility complex, class II, DR beta 5 | 3.866 | transmembrane receptor | Plasma Membrane |
| ANTXRLP1: anthrax toxin receptor-like pseudogene 1 | 3.827 | other | Unknown |

| | | | |
|---|---|---|---|
| PLXNC1: plexin C1 | 3.782 | transmembrane receptor | Plasma Membrane |
| LOC101929310: uncharacterized LOC101929310 | 3.778 | other | Unknown |
| PARVG: parvin, gamma | 3.778 | other | Cytoplasm |
| KIAA1211: KIAA1211 | 3.765 | other | Unknown |
| ZAP70: zeta-chain (TCR) associated protein kinase 70kDa | 3.759 | kinase | Plasma Membrane |
| TRIM59: tripartite motif containing 59 | 3.759 | enzyme | Cytoplasm |
| DENND1C: DENN/MADD domain containing 1C | 3.744 | other | Unknown |
| HAVCR2: hepatitis A virus cellular receptor 2 | 3.714 | other | Plasma Membrane |
| RASGEF1B: RasGEF domain family, member 1B | 3.706 | other | Unknown |
| TLR5: toll-like receptor 5 | 3.7 | transmembrane receptor | Plasma Membrane |
| LOC101929917: uncharacterized LOC101929917 | 3.691 | other | Unknown |
| AIM2: absent in melanoma 2 | 3.675 | other | Cytoplasm |
| SPN: sialophorin | 3.669 | transmembrane receptor | Plasma Membrane |
| PCED1B-AS1: PCED1B antisense RNA 1 | 3.658 | other | Unknown |
| KLRC2: killer cell lectin-like receptor subfamily C, member 2 | 3.657 | transmembrane receptor | Plasma Membrane |
| PLB1: phospholipase B1 | 3.653 | enzyme | Cytoplasm |
| LINC00996: long intergenic non-protein coding RNA 996 | 3.643 | other | Unknown |
| C12orf75: chromosome 12 open reading frame 75 | 3.631 | other | Unknown |
| VNN2: vanin 2 | 3.629 | enzyme | Unknown |
| SIX1: SIX homeobox 1 | 3.629 | transcription regulator | Nucleus |
| BEND4: BEN domain containing 4 | 3.613 | other | Unknown |
| SASH3: SAM and SH3 domain containing 3 | 3.612 | other | Cytoplasm |
| PIK3IP1: phosphoinositide-3-kinase interacting protein 1 | 3.609 | other | Unknown |
| RCAN3: RCAN family member 3 | 3.596 | other | Unknown |
| COL22A1: collagen, type XXII, alpha 1 | 3.594 | other | Extracellular Space |
| ADAMTS14: ADAM metallopeptidase with thrombospondin type 1 motif, 14 | 3.593 | peptidase | Extracellular Space |
| CLEC2D: C-type lectin domain family 2, member D | 3.572 | transmembrane receptor | Plasma Membrane |
| RAB37: RAB37, member RAS oncogene family | 3.562 | enzyme | Cytoplasm |
| LINC00649: long intergenic non-protein coding RNA 649 | 3.559 | other | Unknown |
| RLTPR: RGD motif, leucine rich repeats, tropomodulin domain and proline-rich containing | 3.542 | other | Plasma Membrane |
| HLA-DQA2: major histocompatibility complex, class II, DQ alpha 2 | 3.536 | transmembrane receptor | Plasma Membrane |
| APOBEC3G: apolipoprotein B mRNA editing enzyme, catalytic polypeptide-like 3G | 3.52 | enzyme | Nucleus |
| HLA-DRB1: major histocompatibility complex, class II, DR beta 1 | 3.509 | other | Cytoplasm |
| SLITRK4: SLIT and NTRK-like family, member 4 | 3.504 | other | Extracellular Space |
| TMIGD2: transmembrane and immunoglobulin domain containing 2 | 3.494 | other | Unknown |
| CEACAM21: carcinoembryonic antigen-related cell adhesion molecule 21 | 3.493 | other | Unknown |
| F2RL2: coagulation factor II (thrombin) receptor-like 2 | 3.492 | G-protein coupled recepto | Plasma Membrane |
| ZNF831: zinc finger protein 831 | 3.487 | other | Unknown |

| | | | |
|---|---|---|---|
| BTN3A3: butyrophilin, subfamily 3, member A3 | 3.483 | other | Plasma Membrane |
| SLA: Src-like-adaptor | 3.48 | other | Plasma Membrane |
| BIRC3: baculoviral IAP repeat containing 3 | 3.474 | enzyme | Cytoplasm |
| TDO2: tryptophan 2,3-dioxygenase | 3.467 | enzyme | Cytoplasm |
| CD83: CD83 molecule | 3.466 | transmembrane receptor | Plasma Membrane |
| NOG: noggin | 3.466 | growth factor | Extracellular Space |
| FAM129C: family with sequence similarity 129, member C | 3.46 | other | Unknown |
| NUP210: nucleoporin 210kDa | 3.459 | transporter | Nucleus |
| CLEC6A: C-type lectin domain family 6, member A | 3.457 | transmembrane receptor | Plasma Membrane |
| GLT1D1: glycosyltransferase 1 domain containing 1 | 3.455 | enzyme | Extracellular Space |
| CRISPLD1: cysteine-rich secretory protein LCCL domain containing 1 | 3.448 | other | Cytoplasm |
| TNFRSF13C: tumor necrosis factor receptor superfamily, member 13C | 3.443 | transmembrane receptor | Plasma Membrane |
| TIFAB: TRAF-interacting protein with forkhead-associated domain, family member B | 3.439 | other | Unknown |
| NKG7: natural killer cell granule protein 7 | 3.438 | other | Plasma Membrane |
| TRAF5: TNF receptor-associated factor 5 | 3.437 | transporter | Cytoplasm |
| IL24: interleukin 24 | 3.434 | cytokine | Extracellular Space |
| FGL2: fibrinogen-like 2 | 3.433 | peptidase | Extracellular Space |
| CD5: CD5 molecule | 3.426 | transmembrane receptor | Plasma Membrane |
| SLC14A1: solute carrier family 14 (urea transporter), member 1 (Kidd blood group) | 3.415 | transporter | Plasma Membrane |
| BASP1: brain abundant, membrane attached signal protein 1 | 3.411 | transcription regulator | Nucleus |
| IL18: interleukin 18 | 3.405 | cytokine | Extracellular Space |
| CXCL16: chemokine (C-X-C motif) ligand 16 | 3.397 | cytokine | Extracellular Space |
| AIM1: absent in melanoma 1 | 3.389 | other | Extracellular Space |
| FDCSP: follicular dendritic cell secreted protein | 3.369 | other | Extracellular Space |
| MZB1: marginal zone B and B1 cell-specific protein | 3.368 | other | Extracellular Space |
| CHRM3-AS2: CHRM3 antisense RNA 2 | 3.365 | other | Unknown |
| CORO1A: coronin, actin binding protein, 1A | 3.363 | other | Cytoplasm |
| CLEC4D: C-type lectin domain family 4, member D | 3.359 | other | Plasma Membrane |
| TEX15: testis expressed 15 | 3.351 | other | Extracellular Space |
| IFIT2: interferon-induced protein with tetratricopeptide repeats 2 | 3.347 | other | Cytoplasm |
| PARP15: poly (ADP-ribose) polymerase family, member 15 | 3.347 | other | Unknown |
| ARHGEF18: Rho/Rac guanine nucleotide exchange factor (GEF) 18 | 3.345 | other | Cytoplasm |
| RAC2: ras-related C3 botulinum toxin substrate 2 (rho family, small GTP binding protein Rac | 3.344 | enzyme | Cytoplasm |
| SAMSN1: SAM domain, SH3 domain and nuclear localization signals 1 | 3.34 | other | Nucleus |
| NOX4: NADPH oxidase 4 | 3.34 | enzyme | Cytoplasm |
| TMEM154: transmembrane protein 154 | 3.339 | other | Unknown |
| SIRPB1: signal-regulatory protein beta 1 | 3.339 | other | Plasma Membrane |
| DAZL: deleted in azoospermia-like | 3.336 | translation regulator | Cytoplasm |

| | | | |
|---|---|---|---|
| MXD1: MAX dimerization protein 1 | 3.332 | transcription regulator | Nucleus |
| SH2D2A: SH2 domain containing 2A | 3.331 | other | Cytoplasm |
| XAF1: XIAP associated factor 1 | 3.317 | other | Nucleus |
| ZCCHC12: zinc finger, CCHC domain containing 12 | 3.317 | transcription regulator | Nucleus |
| SNORD3C: small nucleolar RNA, C/D box 3C | 3.306 | other | Unknown |
| C12orf42: chromosome 12 open reading frame 42 | 3.288 | other | Unknown |
| S100Z: S100 calcium binding protein Z | 3.287 | other | Unknown |
| NLRC5: NLR family, CARD domain containing 5 | 3.283 | transcription regulator | Cytoplasm |
| PLXDC2: plexin domain containing 2 | 3.281 | other | Extracellular Space |
| TNFAIP8: tumor necrosis factor, alpha-induced protein 8 | 3.274 | other | Cytoplasm |
| PLA2R1: phospholipase A2 receptor 1, 180kDa | 3.267 | transmembrane receptor | Plasma Membrane |
| HSF5: heat shock transcription factor family member 5 | 3.257 | other | Unknown |
| EOMES: eomesodermin | 3.257 | transcription regulator | Nucleus |
| IFI44: interferon-induced protein 44 | 3.256 | other | Cytoplasm |
| CLDN1: claudin 1 | 3.251 | other | Plasma Membrane |
| BANK1: B-cell scaffold protein with ankyrin repeats 1 | 3.238 | other | Extracellular Space |
| DEF6: differentially expressed in FDCP 6 homolog (mouse) | 3.233 | other | Extracellular Space |
| LOC101928136: uncharacterized LOC101928136 | 3.229 | other | Unknown |
| INPP5D: inositol polyphosphate-5-phosphatase, 145kDa | 3.228 | phosphatase | Cytoplasm |
| LPXN: leupaxin | 3.226 | transcription regulator | Cytoplasm |
| GIMAP2: GTPase, IMAP family member 2 | 3.214 | other | Cytoplasm |
| JAK3: Janus kinase 3 | 3.214 | kinase | Cytoplasm |
| LINC00607: long intergenic non-protein coding RNA 607 | 3.205 | other | Unknown |
| CXCR2: chemokine (C-X-C motif) receptor 2 | 3.201 | G-protein coupled recepto | Plasma Membrane |
| APBA2: amyloid beta (A4) precursor protein-binding, family A, member 2 | 3.198 | transporter | Cytoplasm |
| P2RX7: purinergic receptor P2X, ligand-gated ion channel, 7 | 3.196 | ion channel | Plasma Membrane |
| RGS4: regulator of G-protein signaling 4 | 3.193 | other | Cytoplasm |
| LRRC15: leucine rich repeat containing 15 | 3.189 | other | Plasma Membrane |
| PARP8: poly (ADP-ribose) polymerase family, member 8 | 3.186 | other | Unknown |
| CARD11: caspase recruitment domain family, member 11 | 3.183 | kinase | Cytoplasm |
| CARD16: caspase recruitment domain family, member 16 | 3.176 | other | Unknown |
| PIK3R5: phosphoinositide-3-kinase, regulatory subunit 5 | 3.166 | kinase | Cytoplasm |
| SPINK1: serine peptidase inhibitor, Kazal type 1 | 3.165 | other | Extracellular Space |
| APOBR: apolipoprotein B receptor | 3.163 | transmembrane receptor | Plasma Membrane |
| TLR2: toll-like receptor 2 | 3.162 | transmembrane receptor | Plasma Membrane |
| PATL2: protein associated with topoisomerase II homolog 2 (yeast) | 3.156 | translation regulator | Cytoplasm |
| HOXC8: homeobox C8 | 3.152 | transcription regulator | Nucleus |
| TMC3: transmembrane channel-like 3 | 3.147 | other | Unknown |

| | | | |
|---|---|---|---|
| BGN: biglycan | 3.136 | other | Extracellular Space |
| PTGDR: prostaglandin D2 receptor (DP) | 3.128 | G-protein coupled recepto | Plasma Membrane |
| ANKRD44: ankyrin repeat domain 44 | 3.126 | other | Unknown |
| UBD: ubiquitin D | 3.123 | other | Nucleus |
| WIPF1: WAS/WASL interacting protein family, member 1 | 3.117 | other | Cytoplasm |
| LINC00494: long intergenic non-protein coding RNA 494 | 3.116 | other | Unknown |
| SIDT1: SID1 transmembrane family, member 1 | 3.115 | other | Unknown |
| MYB-AS1: MYB antisense RNA 1 | 3.111 | other | Unknown |
| MAP3K7CL: MAP3K7 C-terminal like | 3.108 | other | Unknown |
| TP53INP1: tumor protein p53 inducible nuclear protein 1 | 3.104 | other | Nucleus |
| AKNA: AT-hook transcription factor | 3.101 | other | Nucleus |
| AMPD3: adenosine monophosphate deaminase 3 | 3.094 | enzyme | Cytoplasm |
| GMIP: GEM interacting protein | 3.088 | enzyme | Cytoplasm |
| CLEC4M: C-type lectin domain family 4, member M | 3.088 | other | Plasma Membrane |
| LINC01222: long intergenic non-protein coding RNA 1222 | 3.085 | other | Unknown |
| GPR171: G protein-coupled receptor 171 | 3.083 | G-protein coupled recepto | Plasma Membrane |
| BLK: B lymphoid tyrosine kinase | 3.077 | kinase | Cytoplasm |
| BCL2L11: BCL2-like 11 (apoptosis facilitator) | 3.073 | other | Cytoplasm |
| HELB: helicase (DNA) B | 3.068 | enzyme | Nucleus |
| DSC1: desmocollin 1 | 3.066 | other | Plasma Membrane |
| LOC101928744: uncharacterized LOC101928744 | 3.066 | other | Unknown |
| PSTPIP2: proline-serine-threonine phosphatase interacting protein 2 | 3.061 | other | Cytoplasm |
| SH2D1B: SH2 domain containing 1B | 3.055 | other | Cytoplasm |
| HLA-DRB6: major histocompatibility complex, class II, DR beta 6 (pseudogene) | 3.051 | other | Unknown |
| HOXC5: homeobox C5 | 3.05 | transcription regulator | Nucleus |
| CNR1: cannabinoid receptor 1 (brain) | 3.026 | G-protein coupled recepto | Plasma Membrane |
| OSM: oncostatin M | 3.017 | cytokine | Extracellular Space |
| KLRD1: killer cell lectin-like receptor subfamily D, member 1 | 3.006 | transmembrane receptor | Plasma Membrane |
| GPSM3: G-protein signaling modulator 3 | 3.002 | other | Cytoplasm |
| KIF21B: kinesin family member 21B | 2.993 | other | Cytoplasm |
| FAM65B: family with sequence similarity 65, member B | 2.99 | other | Unknown |
| REXO1L1P: REX1, RNA exonuclease 1 homolog (S. cerevisiae)-like 1, pseudogene | 2.99 | other | Unknown |
| CLEC2B: C-type lectin domain family 2, member B | 2.969 | other | Plasma Membrane |
| GPR183: G protein-coupled receptor 183 | 2.966 | other | Plasma Membrane |
| HOXA10: homeobox A10 | 2.956 | transcription regulator | Nucleus |
| C9orf66: chromosome 9 open reading frame 66 | 2.943 | other | Unknown |
| MS4A3: membrane-spanning 4-domains, subfamily A, member 3 (hematopoietic cell-specific) | 2.942 | other | Plasma Membrane |
| TIGIT: T cell immunoreceptor with Ig and ITIM domains | 2.941 | other | Plasma Membrane |

| | | | |
|---|---|---|---|
| HIF1A-AS2: HIF1A antisense RNA 2 | 2.935 | other | Unknown |
| THSD7B: thrombospondin, type I, domain containing 7B | 2.935 | other | Unknown |
| ANKRD55: ankyrin repeat domain 55 | 2.935 | transcription regulator | Nucleus |
| TRBV19: T cell receptor beta variable 19 | 2.934 | other | Unknown |
| LOC101929241: uncharacterized LOC101929241 | 2.928 | other | Unknown |
| C1orf162: chromosome 1 open reading frame 162 | 2.924 | transporter | Unknown |
| GLYATL2: glycine-N-acyltransferase-like 2 | 2.922 | other | Unknown |
| DNAH8: dynein, axonemal, heavy chain 8 | 2.913 | enzyme | Cytoplasm |
| MYO1G: myosin IG | 2.901 | other | Cytoplasm |
| SLC27A2: solute carrier family 27 (fatty acid transporter), member 2 | 2.895 | transporter | Cytoplasm |
| CTGF: connective tissue growth factor | 2.895 | growth factor | Extracellular Space |
| JAZF1-AS1: JAZF1 antisense RNA 1 | 2.895 | other | Unknown |
| LPAR2: lysophosphatidic acid receptor 2 | 2.89 | G-protein coupled recepto | Plasma Membrane |
| SLC22A2: solute carrier family 22 (organic cation transporter), member 2 | 2.89 | transporter | Plasma Membrane |
| RASGRP1: RAS guanyl releasing protein 1 (calcium and DAG-regulated) | 2.881 | other | Cytoplasm |
| ARRB2: arrestin, beta 2 | 2.879 | other | Cytoplasm |
| TNFRSF25: tumor necrosis factor receptor superfamily, member 25 | 2.874 | transmembrane receptor | Plasma Membrane |
| PTPN7: protein tyrosine phosphatase, non-receptor type 7 | 2.87 | phosphatase | Cytoplasm |
| HIST1H2AC: histone cluster 1, H2ac | 2.859 | other | Nucleus |
| HSH2D: hematopoietic SH2 domain containing | 2.855 | other | Cytoplasm |
| GRIA2: glutamate receptor, ionotropic, AMPA 2 | 2.854 | ion channel | Plasma Membrane |
| FAM117B: family with sequence similarity 117, member B | 2.852 | other | Unknown |
| PMEL: premelanosome protein | 2.839 | enzyme | Plasma Membrane |
| DLEU7-AS1: DLEU7 antisense RNA 1 | 2.834 | other | Unknown |
| FGD3: FYVE, RhoGEF and PH domain containing 3 | 2.828 | other | Cytoplasm |
| LOC93432: maltase-glucoamylase (alpha-glucosidase) pseudogene | 2.827 | other | Unknown |
| LOC101928443: uncharacterized LOC101928443 | 2.823 | other | Unknown |
| SIGLEC8: sialic acid binding Ig-like lectin 8 | 2.821 | transmembrane receptor | Plasma Membrane |
| KLRG1: killer cell lectin-like receptor subfamily G, member 1 | 2.813 | other | Plasma Membrane |
| IL1RN: interleukin 1 receptor antagonist | 2.81 | cytokine | Extracellular Space |
| PTGS2: prostaglandin-endoperoxide synthase 2 (prostaglandin G/H synthase and cyclooxyge | 2.804 | enzyme | Cytoplasm |
| GCNT4: glucosaminyl (N-acetyl) transferase 4, core 2 | 2.801 | enzyme | Cytoplasm |
| CD5L: CD5 molecule-like | 2.799 | transmembrane receptor | Plasma Membrane |
| COL8A1: collagen, type VIII, alpha 1 | 2.786 | other | Extracellular Space |
| NRK: Nik related kinase | 2.786 | kinase | Unknown |
| HOXC9: homeobox C9 | 2.782 | transcription regulator | Nucleus |
| HLA-F: major histocompatibility complex, class I, F | 2.778 | transmembrane receptor | Plasma Membrane |
| HP09025: uncharacterized LOC100652929 | 2.778 | other | Unknown |

| | | | |
|---|---|---|---|
| TBC1D10C: TBC1 domain family, member 10C | 2.774 | other | Nucleus |
| DLX6-AS1: DLX6 antisense RNA 1 | 2.759 | other | Unknown |
| DEFA1 (includes others): defensin, alpha 1 | 2.759 | other | Cytoplasm |
| CCL22: chemokine (C-C motif) ligand 22 | 2.757 | cytokine | Extracellular Space |
| SMOC2: SPARC related modular calcium binding 2 | 2.757 | other | Extracellular Space |
| ABCA13: ATP-binding cassette, sub-family A (ABC1), member 13 | 2.755 | transporter | Extracellular Space |
| CYP11B1: cytochrome P450, family 11, subfamily B, polypeptide 1 | 2.755 | enzyme | Cytoplasm |
| SYT12: synaptotagmin XII | 2.737 | transporter | Plasma Membrane |
| HOXC10: homeobox C10 | 2.736 | transcription regulator | Nucleus |
| MUC2: mucin 2, oligomeric mucus/gel-forming | 2.734 | other | Unknown |
| PTK2B: protein tyrosine kinase 2 beta | 2.734 | kinase | Cytoplasm |
| MPO: myeloperoxidase | 2.732 | enzyme | Cytoplasm |
| HOXC4: homeobox C4 | 2.725 | transcription regulator | Nucleus |
| LINC00643: long intergenic non-protein coding RNA 643 | 2.717 | other | Unknown |
| MROH9: maestro heat-like repeat family member 9 | 2.712 | other | Unknown |
| LIPM: lipase, family member M | 2.701 | enzyme | Extracellular Space |
| PIK3CD: phosphatidylinositol-4,5-bisphosphate 3-kinase, catalytic subunit delta | 2.686 | kinase | Cytoplasm |
| CD22: CD22 molecule | 2.685 | transmembrane receptor | Plasma Membrane |
| KIAA1210: KIAA1210 | 2.683 | other | Unknown |
| TDGF1: teratocarcinoma-derived growth factor 1 | 2.681 | growth factor | Extracellular Space |
| SSTR3: somatostatin receptor 3 | 2.676 | G-protein coupled recepto | Plasma Membrane |
| SIDT1-AS1: SIDT1 antisense RNA 1 | 2.665 | other | Unknown |
| CLC: Charcot-Leyden crystal galectin | 2.639 | enzyme | Cytoplasm |
| C15orf53: chromosome 15 open reading frame 53 | 2.633 | other | Unknown |
| DGKA: diacylglycerol kinase, alpha 80kDa | 2.632 | kinase | Cytoplasm |
| PZP: pregnancy-zone protein | 2.628 | other | Extracellular Space |
| EDIL3: EGF-like repeats and discoidin I-like domains 3 | 2.628 | other | Extracellular Space |
| SPOCK3: sparc/osteonectin, cwcv and kazal-like domains proteoglycan (testican) 3 | 2.621 | other | Extracellular Space |
| IGKV2-24: immunoglobulin kappa variable 2-24 | 2.618 | other | Unknown |
| CD1E: CD1e molecule | 2.612 | other | Cytoplasm |
| GPCPD1: glycerophosphocholine phosphodiesterase GDE1 homolog (S. cerevisiae) | 2.607 | other | Unknown |
| EVL: Enah/Vasp-like | 2.6 | other | Plasma Membrane |
| LOC101928716: uncharacterized LOC101928716 | 2.6 | other | Unknown |
| GK: glycerol kinase | 2.597 | kinase | Cytoplasm |
| HMHA1: histocompatibility (minor) HA-1 | 2.595 | transporter | Cytoplasm |
| SIT1: signaling threshold regulating transmembrane adaptor 1 | 2.594 | other | Plasma Membrane |
| CCDC88C: coiled-coil domain containing 88C | 2.592 | other | Unknown |
| ITGA11: integrin, alpha 11 | 2.588 | other | Plasma Membrane |

| | | | |
|---|---|---|---|
| SEPT1: septin 1 | 2.572 | enzyme | Cytoplasm |
| SRGN: serglycin | 2.568 | other | Cytoplasm |
| TCF7: transcription factor 7 (T-cell specific, HMG-box) | 2.564 | transcription regulator | Nucleus |
| TCL1A: T-cell leukemia/lymphoma 1A | 2.564 | transcription regulator | Nucleus |
| CD19: CD19 molecule | 2.562 | transmembrane receptor | Plasma Membrane |
| CA6: carbonic anhydrase VI | 2.562 | enzyme | Extracellular Space |
| PRSS3P2: protease, serine, 3 pseudogene 2 | 2.561 | other | Unknown |
| IGHV4-31: immunoglobulin heavy variable 4-31 | 2.55 | other | Unknown |
| LOC100130992: uncharacterized LOC100130992 | 2.537 | other | Unknown |
| LPO: lactoperoxidase | 2.528 | enzyme | Extracellular Space |
| SLC6A1: solute carrier family 6 (neurotransmitter transporter), member 1 | 2.525 | transporter | Plasma Membrane |
| FCER1A: Fc fragment of IgE, high affinity I, receptor for; alpha polypeptide | 2.518 | transmembrane receptor | Plasma Membrane |
| TDRD6: tudor domain containing 6 | 2.517 | other | Cytoplasm |
| CD27: CD27 molecule | 2.514 | transmembrane receptor | Plasma Membrane |
| NLRC3: NLR family, CARD domain containing 3 | 2.512 | other | Cytoplasm |
| STK4: serine/threonine kinase 4 | 2.511 | kinase | Cytoplasm |
| LINC01108: long intergenic non-protein coding RNA 1108 | 2.51 | other | Unknown |
| TFAP2B: transcription factor AP-2 beta (activating enhancer binding protein 2 beta) | 2.509 | transcription regulator | Nucleus |
| HOTAIR: HOX transcript antisense RNA | 2.508 | other | Unknown |
| CXorf65: chromosome X open reading frame 65 | 2.505 | other | Unknown |
| CNR2: cannabinoid receptor 2 (macrophage) | 2.5 | G-protein coupled recepto | Plasma Membrane |
| TRAF1: TNF receptor-associated factor 1 | 2.498 | other | Cytoplasm |
| FCAR: Fc fragment of IgA, receptor for | 2.487 | other | Plasma Membrane |
| PNMA5: paraneoplastic Ma antigen family member 5 | 2.483 | other | Unknown |
| P2RX5: purinergic receptor P2X, ligand-gated ion channel, 5 | 2.474 | ion channel | Plasma Membrane |
| IGLL1/IGLL5: immunoglobulin lambda-like polypeptide 1 | 2.467 | other | Plasma Membrane |
| VNN3: vanin 3 | 2.452 | enzyme | Extracellular Space |
| BCORP1: BCL6 corepressor pseudogene 1 | 2.447 | other | Unknown |
| BARX2: BARX homeobox 2 | 2.447 | transcription regulator | Nucleus |
| C11orf21: chromosome 11 open reading frame 21 | 2.446 | other | Cytoplasm |
| MIR1587: microRNA 1587 | 2.441 | microRNA | Cytoplasm |
| OXTR: oxytocin receptor | 2.426 | G-protein coupled recepto | Plasma Membrane |
| LOC100506393: uncharacterized LOC100506393 | 2.419 | other | Unknown |
| LAMP5: lysosomal-associated membrane protein family, member 5 | 2.409 | other | Cytoplasm |
| C14orf64: chromosome 14 open reading frame 64 | 2.404 | other | Unknown |
| BHLHE41: basic helix-loop-helix family, member e41 | 2.364 | transcription regulator | Nucleus |
| MAS1: MAS1 oncogene | 2.355 | G-protein coupled recepto | Plasma Membrane |
| LOC100130502: uncharacterized LOC100130502 | 2.354 | other | Unknown |

| | | | |
|---|---|---|---|
| LTB: lymphotoxin beta (TNF superfamily, member 3) | 2.345 | cytokine | Extracellular Space |
| SBK1: SH3 domain binding kinase 1 | 2.328 | kinase | Unknown |
| HOXB-AS3: HOXB cluster antisense RNA 3 | 2.32 | other | Unknown |
| CDX2: caudal type homeobox 2 | 2.319 | transcription regulator | Nucleus |
| MST4: serine/threonine protein kinase MST4 | 2.309 | kinase | Nucleus |
| APBB3: amyloid beta (A4) precursor protein-binding, family B, member 3 | 2.306 | other | Cytoplasm |
| MPP1: membrane protein, palmitoylated 1, 55kDa | 2.296 | kinase | Plasma Membrane |
| DEFA4: defensin, alpha 4, corticostatin | 2.274 | other | Extracellular Space |
| APELA: apelin receptor early endogenous ligand | 2.271 | other | Extracellular Space |
| GK-AS1: GK antisense RNA 1 | 2.262 | other | Unknown |
| SLAMF1: signaling lymphocytic activation molecule family member 1 | 2.256 | transmembrane receptor | Plasma Membrane |
| KLRC3: killer cell lectin-like receptor subfamily C, member 3 | 2.245 | transmembrane receptor | Plasma Membrane |
| GNA13: guanine nucleotide binding protein (G protein), alpha 13 | 2.245 | enzyme | Plasma Membrane |
| RPS6KA2-AS1: RPS6KA2 antisense RNA 1 | 2.243 | other | Unknown |
| ANKRD37: ankyrin repeat domain 37 | 2.229 | other | Unknown |
| CPXM2: carboxypeptidase X (M14 family), member 2 | 2.223 | peptidase | Extracellular Space |
| PFKFB4: 6-phosphofructo-2-kinase/fructose-2,6-biphosphatase 4 | 2.222 | kinase | Cytoplasm |
| LINC01141: long intergenic non-protein coding RNA 1141 | 2.22 | other | Unknown |
| A2MP1: alpha-2-macroglobulin pseudogene 1 | 2.216 | other | Unknown |
| HMOX1: heme oxygenase (decycling) 1 | 2.205 | enzyme | Cytoplasm |
| SPIC: Spi-C transcription factor (Spi-1/PU.1 related) | 2.193 | transcription regulator | Nucleus |
| FGF20: fibroblast growth factor 20 | 2.189 | growth factor | Extracellular Space |
| ERRFI1: ERBB receptor feedback inhibitor 1 | 2.186 | other | Cytoplasm |
| GPR128: G protein-coupled receptor 128 | 2.181 | G-protein coupled recepto | Plasma Membrane |
| ITGA8: integrin, alpha 8 | 2.155 | other | Plasma Membrane |
| LOC340113: uncharacterized LOC340113 | 2.147 | other | Unknown |
| CCR7: chemokine (C-C motif) receptor 7 | 2.141 | G-protein coupled recepto | Plasma Membrane |
| SUSD5: sushi domain containing 5 | 2.13 | other | Unknown |
| GPA33: glycoprotein A33 (transmembrane) | 2.121 | other | Plasma Membrane |
| GLB1L3: galactosidase, beta 1-like 3 | 2.114 | enzyme | Unknown |
| CLEC3A: C-type lectin domain family 3, member A | 2.099 | other | Unknown |
| MGAM: maltase-glucoamylase (alpha-glucosidase) | 2.094 | enzyme | Plasma Membrane |
| LINC00243: long intergenic non-protein coding RNA 243 | 2.09 | other | Unknown |
| ATRNL1: attractin-like 1 | 2.081 | other | Unknown |
| NREP-AS1: NREP antisense RNA 1 | 2.078 | other | Unknown |
| LOC101929473: uncharacterized LOC101929473 | 2.068 | other | Unknown |
| SIM1: single-minded family bHLH transcription factor 1 | 2.05 | transcription regulator | Nucleus |
| XRCC6P5: X-ray repair complementing defective repair in Chinese hamster cells 6 pseudoge | 2.044 | other | Unknown |

| | | | |
|---|---|---|---|
| FSHR: follicle stimulating hormone receptor | 2.039 | G-protein coupled recepto | Plasma Membrane |
| OGN: osteoglycin | 2.03 | growth factor | Extracellular Space |
| PAPLN: papilin, proteoglycan-like sulfated glycoprotein | 2.028 | other | Extracellular Space |
| MUC19: mucin 19, oligomeric | 2.024 | other | Cytoplasm |
| LOC101927277: uncharacterized LOC101927277 | 1.989 | other | Unknown |
| HOXC-AS2: HOXC cluster antisense RNA 2 | 1.981 | other | Unknown |
| FLJ45825: uncharacterized LOC100505530 | 1.976 | other | Unknown |
| C5orf17: chromosome 5 open reading frame 17 | 1.974 | other | Unknown |
| C6orf118: chromosome 6 open reading frame 118 | 1.971 | other | Unknown |
| LOC101929734: uncharacterized LOC101929734 | 1.966 | other | Unknown |
| SLC9A2: solute carrier family 9, subfamily A (NHE2, cation proton antiporter 2), member 2 | 1.964 | transporter | Plasma Membrane |
| CNGA4: cyclic nucleotide gated channel alpha 4 | 1.953 | ion channel | Plasma Membrane |
| ASB9: ankyrin repeat and SOCS box containing 9 | 1.948 | transcription regulator | Nucleus |
| TBC1D27: TBC1 domain family, member 27 | 1.902 | other | Unknown |
| LOC101928535: uncharacterized LOC101928535 | 1.883 | other | Unknown |
| PI16: peptidase inhibitor 16 | 1.878 | other | Extracellular Space |
| HAVCR1: hepatitis A virus cellular receptor 1 | 1.868 | other | Plasma Membrane |
| HBG2: hemoglobin, gamma G | 1.858 | other | Cytoplasm |
| LOC101928773: uncharacterized LOC101928773 | 1.855 | other | Unknown |
| TRPV5: transient receptor potential cation channel, subfamily V, member 5 | 1.853 | ion channel | Plasma Membrane |
| LINC01210: long intergenic non-protein coding RNA 1210 | 1.853 | other | Unknown |
| SLC17A8: solute carrier family 17 (vesicular glutamate transporter), member 8 | 1.853 | transporter | Plasma Membrane |
| SIX6: SIX homeobox 6 | 1.838 | transcription regulator | Nucleus |
| LINC01281: long intergenic non-protein coding RNA 1281 | 1.836 | other | Unknown |
| PCDH10: protocadherin 10 | 1.835 | other | Plasma Membrane |
| RASGRP2: RAS guanyl releasing protein 2 (calcium and DAG-regulated) | 1.834 | other | Cytoplasm |
| ANKRD30BL: ankyrin repeat domain 30B-like | 1.828 | other | Unknown |
| TNFRSF13B: tumor necrosis factor receptor superfamily, member 13B | 1.827 | transmembrane receptor | Plasma Membrane |
| EIF4E1B: eukaryotic translation initiation factor 4E family member 1B | 1.822 | other | Unknown |
| CXCR1: chemokine (C-X-C motif) receptor 1 | 1.817 | G-protein coupled recepto | Plasma Membrane |
| DMRT3: doublesex and mab-3 related transcription factor 3 | 1.809 | transcription regulator | Nucleus |
| LOC101928075: uncharacterized LOC101928075 | 1.797 | other | Unknown |
| LOC101928622: uncharacterized LOC101928622 | 1.794 | other | Unknown |
| GABRR3: gamma-aminobutyric acid (GABA) A receptor, rho 3 | 1.791 | transmembrane receptor | Plasma Membrane |
| CCDC42: coiled-coil domain containing 42 | 1.789 | other | Unknown |
| KRT73-AS1: KRT73 antisense RNA 1 | 1.785 | other | Unknown |
| SDS: serine dehydratase | 1.744 | enzyme | Cytoplasm |
| IBSP: integrin-binding sialoprotein | 1.744 | other | Extracellular Space |

| | | | |
|---|---|---|---|
| LINC01020: long intergenic non-protein coding RNA 1020 | 1.738 | other | Unknown |
| DMBT1P1: deleted in malignant brain tumors 1 pseudogene 1 | 1.738 | other | Unknown |
| C1QTNF9B-AS1: C1QTNF9B antisense RNA 1 | 1.726 | other | Cytoplasm |
| HBB: hemoglobin, beta | 1.721 | transporter | Cytoplasm |
| TEX11: testis expressed 11 | 1.718 | other | Nucleus |
| MAGEB18: melanoma antigen family B, 18 | 1.716 | other | Cytoplasm |
| EREG: epiregulin | 1.716 | growth factor | Extracellular Space |
| LOC101928709: uncharacterized LOC101928709 | 1.708 | other | Unknown |
| GP2: glycoprotein 2 (zymogen granule membrane) | 1.707 | other | Cytoplasm |
| MT1H: metallothionein 1H | 1.692 | other | Unknown |
| HOXC12: homeobox C12 | 1.681 | transcription regulator | Nucleus |
| NALCN: sodium leak channel, non-selective | 1.628 | ion channel | Plasma Membrane |
| SPRR3: small proline-rich protein 3 | 1.628 | other | Cytoplasm |
| OLFM4: olfactomedin 4 | 1.609 | other | Extracellular Space |
| MMP27: matrix metallopeptidase 27 | 1.599 | peptidase | Unknown |
| C1orf110: chromosome 1 open reading frame 110 | 1.598 | other | Unknown |
| GABRA1: gamma-aminobutyric acid (GABA) A receptor, alpha 1 | 1.595 | ion channel | Plasma Membrane |
| EPPIN: epididymal peptidase inhibitor | 1.594 | other | Extracellular Space |
| F13B: coagulation factor XIII, B polypeptide | 1.563 | enzyme | Extracellular Space |
| HCRTR2: hypocretin (orexin) receptor 2 | 1.563 | G-protein coupled recepto | Plasma Membrane |
| LINC01203: long intergenic non-protein coding RNA 1203 | 1.559 | other | Unknown |
| SYNPR: synaptoporin | 1.544 | transporter | Plasma Membrane |
| PGC: progastricsin (pepsinogen C) | -1.502 | peptidase | Extracellular Space |
| SCNN1A: sodium channel, non-voltage-gated 1 alpha subunit | -1.503 | ion channel | Plasma Membrane |
| CTSE: cathepsin E | -1.507 | peptidase | Cytoplasm |
| EPCAM: epithelial cell adhesion molecule | -1.51 | other | Plasma Membrane |
| DUOXA1: dual oxidase maturation factor 1 | -1.529 | other | Plasma Membrane |
| TMC5: transmembrane channel-like 5 | -1.54 | other | Unknown |
| CACNA2D2: calcium channel, voltage-dependent, alpha 2/delta subunit 2 | -1.553 | ion channel | Plasma Membrane |
| ATP13A4: ATPase type 13A4 | -1.563 | transporter | Unknown |
| KRT4: keratin 4 | -1.567 | other | Cytoplasm |
| TMEM125: transmembrane protein 125 | -1.567 | other | Unknown |
| AP1M2: adaptor-related protein complex 1, mu 2 subunit | -1.584 | transporter | Cytoplasm |
| LRP2: low density lipoprotein receptor-related protein 2 | -1.604 | transporter | Plasma Membrane |
| CHI3L2: chitinase 3-like 2 | -1.606 | enzyme | Extracellular Space |
| GPRIN2: G protein regulated inducer of neurite outgrowth 2 | -1.61 | other | Unknown |
| BMP10: bone morphogenetic protein 10 | -1.633 | growth factor | Extracellular Space |
| PLIN1: perilipin 1 | -1.633 | other | Cytoplasm |

| | | | |
|---|---|---|---|
| ITGB6: integrin, beta 6 | -1.638 | other | Plasma Membrane |
| BMP3: bone morphogenetic protein 3 | -1.64 | growth factor | Extracellular Space |
| VEPH1: ventricular zone expressed PH domain-containing 1 | -1.641 | other | Nucleus |
| PLA2G1B: phospholipase A2, group IB (pancreas) | -1.685 | enzyme | Extracellular Space |
| CDH1: cadherin 1, type 1, E-cadherin (epithelial) | -1.688 | other | Plasma Membrane |
| SCN1A: sodium channel, voltage-gated, type I, alpha subunit | -1.715 | ion channel | Plasma Membrane |
| ADIPOQ: adiponectin, C1Q and collagen domain containing | -1.722 | other | Extracellular Space |
| DRD5: dopamine receptor D5 | -1.731 | G-protein coupled recepto | Plasma Membrane |
| SHANK2: SH3 and multiple ankyrin repeat domains 2 | -1.754 | other | Plasma Membrane |
| HAS2: hyaluronan synthase 2 | -1.755 | enzyme | Plasma Membrane |
| KLF5: Kruppel-like factor 5 (intestinal) | -1.76 | transcription regulator | Nucleus |
| HRASLS5: HRAS-like suppressor family, member 5 | -1.764 | other | Unknown |
| EXPH5: exophilin 5 | -1.769 | other | Cytoplasm |
| CLDN11: claudin 11 | -1.776 | other | Plasma Membrane |
| NPY2R: neuropeptide Y receptor Y2 | -1.795 | G-protein coupled recepto | Plasma Membrane |
| MISP: mitotic spindle positioning | -1.798 | other | Unknown |
| SFTA3: surfactant associated 3 | -1.8 | other | Unknown |
| CYP2B7P: cytochrome P450, family 2, subfamily B, polypeptide 7, pseudogene | -1.8 | enzyme | Unknown |
| KIAA1244: KIAA1244 | -1.805 | other | Unknown |
| MGC27382: uncharacterized MGC27382 | -1.819 | other | Unknown |
| SDR16C5: short chain dehydrogenase/reductase family 16C, member 5 | -1.824 | enzyme | Cytoplasm |
| CCL2: chemokine (C-C motif) ligand 2 | -1.825 | cytokine | Extracellular Space |
| MLPH: melanophilin | -1.833 | other | Cytoplasm |
| LAMB3: laminin, beta 3 | -1.846 | transporter | Extracellular Space |
| PCSK9: proprotein convertase subtilisin/kexin type 9 | -1.848 | peptidase | Extracellular Space |
| LEP: leptin | -1.852 | growth factor | Extracellular Space |
| MFSD2A: major facilitator superfamily domain containing 2A | -1.863 | other | Unknown |
| FOXA1: forkhead box A1 | -1.873 | transcription regulator | Nucleus |
| AGER: advanced glycosylation end product-specific receptor | -1.875 | transmembrane receptor | Plasma Membrane |
| LOC101929612: uncharacterized LOC101929612 | -1.879 | other | Unknown |
| CPB2: carboxypeptidase B2 (plasma) | -1.885 | peptidase | Extracellular Space |
| SLPI: secretory leukocyte peptidase inhibitor | -1.887 | other | Cytoplasm |
| RAP1GAP: RAP1 GTPase activating protein | -1.917 | other | Cytoplasm |
| NIPAL4: NIPA-like domain containing 4 | -1.919 | other | Unknown |
| MYL1: myosin, light chain 1, alkali; skeletal, fast | -1.92 | other | Cytoplasm |
| AQP4: aquaporin 4 | -1.934 | transporter | Plasma Membrane |
| LTK: leukocyte receptor tyrosine kinase | -1.949 | kinase | Plasma Membrane |
| FAM83E: family with sequence similarity 83, member E | -1.959 | other | Unknown |

| | | | |
|---|---|---|---|
| KLB: klotho beta | -1.96 | enzyme | Plasma Membrane |
| LOC286189: uncharacterized LOC286189 | -1.961 | other | Unknown |
| FNDC4: fibronectin type III domain containing 4 | -1.969 | other | Unknown |
| FGFR2: fibroblast growth factor receptor 2 | -1.986 | kinase | Plasma Membrane |
| OAF: OAF homolog (Drosophila) | -1.99 | other | Unknown |
| TMEM100: transmembrane protein 100 | -2.004 | other | Unknown |
| LGALS12: lectin, galactoside-binding, soluble, 12 | -2.004 | other | Extracellular Space |
| EHF: ets homologous factor | -2.008 | transcription regulator | Nucleus |
| MT-TF: tRNA | -2.013 | other | Cytoplasm |
| MAL2: mal, T-cell differentiation protein 2 (gene/pseudogene) | -2.038 | transporter | Plasma Membrane |
| NMB: neuromedin B | -2.046 | other | Extracellular Space |
| LINC00319: long intergenic non-protein coding RNA 319 | -2.07 | other | Unknown |
| LIPH: lipase, member H | -2.073 | enzyme | Extracellular Space |
| ACKR1: atypical chemokine receptor 1 (Duffy blood group) | -2.083 | G-protein coupled recepto | Plasma Membrane |
| MET: met proto-oncogene | -2.114 | kinase | Plasma Membrane |
| GPAM: glycerol-3-phosphate acyltransferase, mitochondrial | -2.126 | enzyme | Cytoplasm |
| RHBDL2: rhomboid, veinlet-like 2 (Drosophila) | -2.147 | peptidase | Plasma Membrane |
| CHIAP2: chitinase, acidic pseudogene 2 | -2.157 | other | Unknown |
| FRMD1: FERM domain containing 1 | -2.158 | other | Unknown |
| DNAJA4: DnaJ (Hsp40) homolog, subfamily A, member 4 | -2.159 | other | Nucleus |
| LIPG: lipase, endothelial | -2.173 | enzyme | Extracellular Space |
| ID4: inhibitor of DNA binding 4, dominant negative helix-loop-helix protein | -2.179 | transcription regulator | Nucleus |
| C1orf116: chromosome 1 open reading frame 116 | -2.181 | other | Cytoplasm |
| VIPR1: vasoactive intestinal peptide receptor 1 | -2.208 | G-protein coupled recepto | Plasma Membrane |
| FN3K: fructosamine 3 kinase | -2.218 | kinase | Cytoplasm |
| AGR2: anterior gradient 2 | -2.221 | other | Extracellular Space |
| SLC6A14: solute carrier family 6 (amino acid transporter), member 14 | -2.25 | transporter | Plasma Membrane |
| HMGCS2: 3-hydroxy-3-methylglutaryl-CoA synthase 2 (mitochondrial) | -2.259 | enzyme | Cytoplasm |
| RORC: RAR-related orphan receptor C | -2.265 | ligand-dependent nuclear | Nucleus |
| NAPSA: napsin A aspartic peptidase | -2.27 | peptidase | Extracellular Space |
| SFTPC: surfactant protein C | -2.272 | other | Extracellular Space |
| ESRP1: epithelial splicing regulatory protein 1 | -2.272 | other | Nucleus |
| PLXNA2: plexin A2 | -2.276 | transmembrane receptor | Plasma Membrane |
| CAPN8: calpain 8 | -2.297 | peptidase | Cytoplasm |
| EPS8L2: EPS8-like 2 | -2.308 | other | Unknown |
| FXYD3: FXYD domain containing ion transport regulator 3 | -2.308 | other | Plasma Membrane |
| DUOX1: dual oxidase 1 | -2.313 | enzyme | Plasma Membrane |
| RBP4: retinol binding protein 4, plasma | -2.321 | transporter | Extracellular Space |

| | | | |
|---|---|---|---|
| WWC1: WW and C2 domain containing 1 | -2.349 | transcription regulator | Cytoplasm |
| ADAMTS19: ADAM metallopeptidase with thrombospondin type 1 motif, 19 | -2.351 | peptidase | Extracellular Space |
| LPIN1: lipin 1 | -2.36 | phosphatase | Nucleus |
| SFTPB: surfactant protein B | -2.362 | other | Extracellular Space |
| GRAMD2: GRAM domain containing 2 | -2.373 | other | Unknown |
| TMPRSS2: transmembrane protease, serine 2 | -2.378 | peptidase | Plasma Membrane |
| AGPAT9: 1-acylglycerol-3-phosphate O-acyltransferase 9 | -2.379 | enzyme | Cytoplasm |
| ACADL: acyl-CoA dehydrogenase, long chain | -2.382 | enzyme | Cytoplasm |
| PCK1: phosphoenolpyruvate carboxykinase 1 (soluble) | -2.393 | kinase | Cytoplasm |
| BOK: BCL2-related ovarian killer | -2.393 | other | Cytoplasm |
| LRRC75A: leucine rich repeat containing 75A | -2.4 | other | Unknown |
| PPRC1: peroxisome proliferator-activated receptor gamma, coactivator-related 1 | -2.404 | transcription regulator | Nucleus |
| CKMT1A/CKMT1B: creatine kinase, mitochondrial 1B | -2.412 | kinase | Cytoplasm |
| ADAMTS15: ADAM metallopeptidase with thrombospondin type 1 motif, 15 | -2.413 | peptidase | Extracellular Space |
| CYP2B6: cytochrome P450, family 2, subfamily B, polypeptide 6 | -2.421 | enzyme | Cytoplasm |
| CLMP: CXADR-like membrane protein | -2.428 | other | Plasma Membrane |
| SFTA2: surfactant associated 2 | -2.434 | other | Unknown |
| LRRC14B: leucine rich repeat containing 14B | -2.438 | other | Unknown |
| DMBT1: deleted in malignant brain tumors 1 | -2.445 | transmembrane receptor | Plasma Membrane |
| CYP4B1: cytochrome P450, family 4, subfamily B, polypeptide 1 | -2.46 | enzyme | Cytoplasm |
| SHH: sonic hedgehog | -2.462 | peptidase | Extracellular Space |
| GPD1: glycerol-3-phosphate dehydrogenase 1 (soluble) | -2.465 | enzyme | Cytoplasm |
| ACACA: acetyl-CoA carboxylase alpha | -2.472 | enzyme | Cytoplasm |
| DOT1L: DOT1-like histone H3K79 methyltransferase | -2.474 | phosphatase | Nucleus |
| RRP12: ribosomal RNA processing 12 homolog (S. cerevisiae) | -2.476 | other | Nucleus |
| ECHS1: enoyl CoA hydratase, short chain, 1, mitochondrial | -2.477 | enzyme | Cytoplasm |
| FADS1: fatty acid desaturase 1 | -2.48 | enzyme | Plasma Membrane |
| NUDT4: nudix (nucleoside diphosphate linked moiety X)-type motif 4 | -2.493 | phosphatase | Cytoplasm |
| TF: transferrin | -2.494 | transporter | Extracellular Space |
| PLA2G2A: phospholipase A2, group IIA (platelets, synovial fluid) | -2.495 | enzyme | Cytoplasm |
| BAG3: BCL2-associated athanogene 3 | -2.5 | other | Cytoplasm |
| PIGR: polymeric immunoglobulin receptor | -2.506 | transporter | Plasma Membrane |
| PLA2G4F: phospholipase A2, group IVF | -2.511 | enzyme | Cytoplasm |
| AKR1C1/AKR1C2: aldo-keto reductase family 1, member C2 | -2.52 | enzyme | Cytoplasm |
| C10orf62: chromosome 10 open reading frame 62 | -2.525 | other | Unknown |
| MT1M: metallothionein 1M | -2.532 | other | Unknown |
| MYH7B: myosin, heavy chain 7B, cardiac muscle, beta | -2.548 | other | Unknown |
| ETNPPL: ethanolamine-phosphate phospho-lyase | -2.557 | enzyme | Unknown |

| | | | |
|---|---|---|---|
| DGCR6/LOC102724770: DiGeorge syndrome critical region gene 6 | -2.567 | other | Nucleus |
| SNX22: sorting nexin 22 | -2.573 | transporter | Unknown |
| CSF3: colony stimulating factor 3 (granulocyte) | -2.579 | cytokine | Extracellular Space |
| TFPI2: tissue factor pathway inhibitor 2 | -2.58 | other | Extracellular Space |
| ARTN: artemin | -2.585 | growth factor | Extracellular Space |
| NR4A3: nuclear receptor subfamily 4, group A, member 3 | -2.59 | ligand-dependent nuclear | Nucleus |
| LBP: lipopolysaccharide binding protein | -2.591 | transporter | Plasma Membrane |
| DNAJB5: DnaJ (Hsp40) homolog, subfamily B, member 5 | -2.602 | other | Cytoplasm |
| LONRF2: LON peptidase N-terminal domain and ring finger 2 | -2.624 | other | Unknown |
| RSPO4: R-spondin 4 | -2.629 | other | Plasma Membrane |
| ITGB1BP2: integrin beta 1 binding protein (melusin) 2 | -2.633 | other | Unknown |
| SCEL: sciellin | -2.646 | other | Cytoplasm |
| SLC19A2: solute carrier family 19 (thiamine transporter), member 2 | -2.646 | transporter | Plasma Membrane |
| TESC: tescalcin | -2.654 | other | Cytoplasm |
| LOC100507033: uncharacterized LOC100507033 | -2.657 | other | Unknown |
| MT-TP: tRNA | -2.663 | other | Cytoplasm |
| MYL4: myosin, light chain 4, alkali; atrial, embryonic | -2.676 | other | Cytoplasm |
| PEG10: paternally expressed 10 | -2.677 | other | Nucleus |
| KRBA1: KRAB-A domain containing 1 | -2.681 | other | Unknown |
| FILIP1: filamin A interacting protein 1 | -2.682 | other | Cytoplasm |
| PNPLA3: patatin-like phospholipase domain containing 3 | -2.687 | enzyme | Cytoplasm |
| COL4A4: collagen, type IV, alpha 4 | -2.691 | other | Extracellular Space |
| F11: coagulation factor XI | -2.699 | peptidase | Extracellular Space |
| ART3: ADP-ribosyltransferase 3 | -2.714 | enzyme | Plasma Membrane |
| SYT7: synaptotagmin VII | -2.717 | transporter | Cytoplasm |
| C3orf52: chromosome 3 open reading frame 52 | -2.718 | other | Unknown |
| MLXIPL: MLX interacting protein-like | -2.72 | transcription regulator | Nucleus |
| BDKRB2: bradykinin receptor B2 | -2.723 | G-protein coupled recepto | Plasma Membrane |
| HOGA1: 4-hydroxy-2-oxoglutarate aldolase 1 | -2.723 | enzyme | Cytoplasm |
| GAREM: GRB2 associated, regulator of MAPK1 | -2.728 | other | Unknown |
| THRSP: thyroid hormone responsive | -2.733 | other | Nucleus |
| DHCR7: 7-dehydrocholesterol reductase | -2.739 | enzyme | Cytoplasm |
| SELENBP1: selenium binding protein 1 | -2.762 | other | Cytoplasm |
| RBM24: RNA binding motif protein 24 | -2.778 | other | Unknown |
| CADM4: cell adhesion molecule 4 | -2.782 | other | Plasma Membrane |
| DGKD: diacylglycerol kinase, delta 130kDa | -2.785 | kinase | Cytoplasm |
| ALPL: alkaline phosphatase, liver/bone/kidney | -2.788 | phosphatase | Plasma Membrane |
| SHC2: SHC (Src homology 2 domain containing) transforming protein 2 | -2.791 | other | Cytoplasm |

| | | | |
|---|---|---|---|
| SLC6A4: solute carrier family 6 (neurotransmitter transporter), member 4 | -2.795 | transporter | Plasma Membrane |
| PNPLA2: patatin-like phospholipase domain containing 2 | -2.812 | enzyme | Cytoplasm |
| GPR133: G protein-coupled receptor 133 | -2.816 | G-protein coupled recepto | Plasma Membrane |
| WIF1: WNT inhibitory factor 1 | -2.823 | other | Extracellular Space |
| PVR: poliovirus receptor | -2.825 | other | Plasma Membrane |
| MIDN: midnolin | -2.825 | other | Nucleus |
| KIF1A: kinesin family member 1A | -2.829 | other | Cytoplasm |
| S1PR2: sphingosine-1-phosphate receptor 2 | -2.853 | G-protein coupled recepto | Plasma Membrane |
| MSLN: mesothelin | -2.862 | other | Extracellular Space |
| DGAT2: diacylglycerol O-acyltransferase 2 | -2.863 | enzyme | Cytoplasm |
| GATA2: GATA binding protein 2 | -2.871 | transcription regulator | Nucleus |
| PTPRS: protein tyrosine phosphatase, receptor type, S | -2.883 | phosphatase | Plasma Membrane |
| CIDEC: cell death-inducing DFFA-like effector c | -2.886 | other | Cytoplasm |
| ETFB: electron-transfer-flavoprotein, beta polypeptide | -2.901 | transporter | Cytoplasm |
| SAA2: serum amyloid A2 | -2.907 | other | Extracellular Space |
| SPNS2: spinster homolog 2 (Drosophila) | -2.909 | transporter | Unknown |
| CISH: cytokine inducible SH2-containing protein | -2.92 | other | Cytoplasm |
| PHACTR1: phosphatase and actin regulator 1 | -2.929 | other | Cytoplasm |
| LRRC2: leucine rich repeat containing 2 | -2.932 | other | Unknown |
| TIMP4: TIMP metallopeptidase inhibitor 4 | -2.949 | other | Extracellular Space |
| FGFR4: fibroblast growth factor receptor 4 | -2.951 | kinase | Plasma Membrane |
| NDUFB10: NADH dehydrogenase (ubiquinone) 1 beta subcomplex, 10, 22kDa | -2.954 | enzyme | Cytoplasm |
| TMEM132B: transmembrane protein 132B | -2.962 | other | Unknown |
| KLHL25: kelch-like family member 25 | -2.967 | other | Cytoplasm |
| TGM2: transglutaminase 2 | -3.022 | enzyme | Cytoplasm |
| WISP2: WNT1 inducible signaling pathway protein 2 | -3.028 | growth factor | Extracellular Space |
| HAMP: hepcidin antimicrobial peptide | -3.031 | other | Extracellular Space |
| AGPAT2: 1-acylglycerol-3-phosphate O-acyltransferase 2 | -3.034 | enzyme | Cytoplasm |
| PAPPA: pregnancy-associated plasma protein A, pappalysin 1 | -3.034 | peptidase | Extracellular Space |
| HP: haptoglobin | -3.041 | peptidase | Extracellular Space |
| HHATL: hedgehog acyltransferase-like | -3.042 | enzyme | Cytoplasm |
| SRPX: sushi-repeat containing protein, X-linked | -3.046 | other | Cytoplasm |
| EYA4: eyes absent homolog 4 (Drosophila) | -3.089 | phosphatase | Cytoplasm |
| MAP3K6: mitogen-activated protein kinase kinase kinase 6 | -3.09 | kinase | Unknown |
| KRT19: keratin 19 | -3.092 | other | Cytoplasm |
| ADAMTS9: ADAM metallopeptidase with thrombospondin type 1 motif, 9 | -3.096 | peptidase | Extracellular Space |
| CA3: carbonic anhydrase III, muscle specific | -3.109 | enzyme | Cytoplasm |
| ABCA3: ATP-binding cassette, sub-family A (ABC1), member 3 | -3.115 | transporter | Plasma Membrane |

| | | | |
|---|---|---|---|
| SELE: selectin E | -3.125 | transmembrane receptor | Plasma Membrane |
| GRAMD4: GRAM domain containing 4 | -3.13 | other | Unknown |
| SCD: stearoyl-CoA desaturase (delta-9-desaturase) | -3.139 | enzyme | Cytoplasm |
| TOMM40: translocase of outer mitochondrial membrane 40 homolog (yeast) | -3.161 | ion channel | Cytoplasm |
| MYOC: myocilin, trabecular meshwork inducible glucocorticoid response | -3.164 | other | Cytoplasm |
| C1QTNF1: C1q and tumor necrosis factor related protein 1 | -3.165 | other | Extracellular Space |
| FAM213A: family with sequence similarity 213, member A | -3.171 | other | Extracellular Space |
| A4GALT: alpha 1,4-galactosyltransferase | -3.177 | enzyme | Cytoplasm |
| P2RY2: purinergic receptor P2Y, G-protein coupled, 2 | -3.191 | G-protein coupled recepto | Plasma Membrane |
| ELOVL6: ELOVL fatty acid elongase 6 | -3.193 | enzyme | Cytoplasm |
| RMST: rhabdomyosarcoma 2 associated transcript (non-protein coding) | -3.194 | other | Unknown |
| RBM20: RNA binding motif protein 20 | -3.205 | other | Nucleus |
| COL4A3: collagen, type IV, alpha 3 (Goodpasture antigen) | -3.206 | other | Extracellular Space |
| SYNM: synemin, intermediate filament protein | -3.213 | other | Cytoplasm |
| DPF3: D4, zinc and double PHD fingers, family 3 | -3.215 | other | Unknown |
| AASS: aminoadipate-semialdehyde synthase | -3.24 | enzyme | Cytoplasm |
| CECR2: cat eye syndrome chromosome region, candidate 2 | -3.249 | other | Nucleus |
| TMOD1: tropomodulin 1 | -3.259 | enzyme | Cytoplasm |
| DAPK3: death-associated protein kinase 3 | -3.261 | kinase | Cytoplasm |
| CGN: cingulin | -3.268 | other | Plasma Membrane |
| OBSL1: obscurin-like 1 | -3.273 | other | Cytoplasm |
| RGR: retinal G protein coupled receptor | -3.282 | G-protein coupled recepto | Plasma Membrane |
| SLCO5A1: solute carrier organic anion transporter family, member 5A1 | -3.284 | transporter | Unknown |
| ADAMTS8: ADAM metallopeptidase with thrombospondin type 1 motif, 8 | -3.29 | peptidase | Extracellular Space |
| PRX: periaxin | -3.327 | other | Nucleus |
| SFTPA2: surfactant protein A2 | -3.328 | other | Extracellular Space |
| CYB5A: cytochrome b5 type A (microsomal) | -3.344 | enzyme | Cytoplasm |
| FRAS1: Fraser syndrome 1 | -3.372 | other | Extracellular Space |
| USP54: ubiquitin specific peptidase 54 | -3.376 | peptidase | Unknown |
| SCGN: secretagogin, EF-hand calcium binding protein | -3.423 | other | Cytoplasm |
| SFTPA1: surfactant protein A1 | -3.428 | transporter | Extracellular Space |
| CMTM5: CKLF-like MARVEL transmembrane domain containing 5 | -3.43 | cytokine | Unknown |
| COX5B: cytochrome c oxidase subunit Vb | -3.445 | enzyme | Cytoplasm |
| SOX9: SRY (sex determining region Y)-box 9 | -3.448 | transcription regulator | Nucleus |
| KLHL41: kelch-like family member 41 | -3.458 | other | Cytoplasm |
| PLIN4: perilipin 4 | -3.463 | other | Cytoplasm |
| CFD: complement factor D (adipsin) | -3.481 | peptidase | Extracellular Space |
| FLJ42969: uncharacterized LOC441374 | -3.512 | other | Unknown |

| | | | |
|---|---|---|---|
| SLC34A2: solute carrier family 34 (type II sodium/phosphate contransporter), member 2 | -3.516 | transporter | Plasma Membrane |
| RHOBTB2: Rho-related BTB domain containing 2 | -3.579 | enzyme | Unknown |
| YBX2: Y box binding protein 2 | -3.597 | translation regulator | Cytoplasm |
| HOPX: HOP homeobox | -3.629 | transcription regulator | Nucleus |
| ACADS: acyl-CoA dehydrogenase, C-2 to C-3 short chain | -3.63 | enzyme | Cytoplasm |
| DHCR24: 24-dehydrocholesterol reductase | -3.631 | enzyme | Cytoplasm |
| ADRB1: adrenoceptor beta 1 | -3.637 | G-protein coupled recepto | Plasma Membrane |
| QRFPR: pyroglutamylated RFamide peptide receptor | -3.643 | G-protein coupled recepto | Plasma Membrane |
| CDC42EP4: CDC42 effector protein (Rho GTPase binding) 4 | -3.643 | other | Cytoplasm |
| ANK3: ankyrin 3, node of Ranvier (ankyrin G) | -3.652 | other | Plasma Membrane |
| ACSS2: acyl-CoA synthetase short-chain family member 2 | -3.664 | enzyme | Cytoplasm |
| LARGE: like-glycosyltransferase | -3.67 | enzyme | Cytoplasm |
| ACTA1: actin, alpha 1, skeletal muscle | -3.703 | other | Cytoplasm |
| PPP1R1A: protein phosphatase 1, regulatory (inhibitor) subunit 1A | -3.732 | phosphatase | Cytoplasm |
| MYH8: myosin, heavy chain 8, skeletal muscle, perinatal | -3.761 | enzyme | Cytoplasm |
| LOC102724788/PRODH: proline dehydrogenase (oxidase) 1 | -3.765 | enzyme | Cytoplasm |
| LOC101929335: uncharacterized LOC101929335 | -3.795 | other | Unknown |
| AKAP6: A kinase (PRKA) anchor protein 6 | -3.844 | other | Nucleus |
| FAM160A1: family with sequence similarity 160, member A1 | -3.854 | other | Unknown |
| CALB2: calbindin 2 | -3.868 | other | Cytoplasm |
| PACSIN3: protein kinase C and casein kinase substrate in neurons 3 | -3.919 | other | Cytoplasm |
| CLUH: clustered mitochondria (cluA/CLU1) homolog | -3.923 | translation regulator | Cytoplasm |
| SFTPD: surfactant protein D | -3.95 | other | Extracellular Space |
| CLGN: calmegin | -3.964 | peptidase | Cytoplasm |
| RARRES1: retinoic acid receptor responder (tazarotene induced) 1 | -3.977 | other | Plasma Membrane |
| RTP3: receptor (chemosensory) transporter protein 3 | -3.98 | other | Cytoplasm |
| PCDH9: protocadherin 9 | -4.019 | other | Plasma Membrane |
| CASZ1: castor zinc finger 1 | -4.021 | enzyme | Nucleus |
| CES1: carboxylesterase 1 | -4.068 | enzyme | Cytoplasm |
| CRYAB: crystallin, alpha B | -4.114 | other | Nucleus |
| OPLAH: 5-oxoprolinase (ATP-hydrolysing) | -4.132 | enzyme | Cytoplasm |
| MOG: myelin oligodendrocyte glycoprotein | -4.209 | other | Extracellular Space |
| KCNIP2: Kv channel interacting protein 2 | -4.22 | other | Cytoplasm |
| CORO6: coronin 6 | -4.23 | other | Extracellular Space |
| LMO3: LIM domain only 3 (rhombotin-like 2) | -4.231 | other | Nucleus |
| PLIN5: perilipin 5 | -4.236 | other | Plasma Membrane |
| ALDH1L1: aldehyde dehydrogenase 1 family, member L1 | -4.236 | enzyme | Cytoplasm |
| DUSP27: dual specificity phosphatase 27 (putative) | -4.239 | phosphatase | Unknown |

| | | | |
|---|---|---|---|
| TAC1: tachykinin, precursor 1 | -4.242 | other | Extracellular Space |
| MYO7B: myosin VIIB | -4.26 | peptidase | Unknown |
| CHI3L1: chitinase 3-like 1 (cartilage glycoprotein-39) | -4.274 | enzyme | Extracellular Space |
| RAMP1: receptor (G protein-coupled) activity modifying protein 1 | -4.288 | transporter | Plasma Membrane |
| COBL: cordon-bleu WH2 repeat protein | -4.302 | other | Plasma Membrane |
| C15orf41: chromosome 15 open reading frame 41 | -4.375 | other | Unknown |
| GOT1: glutamic-oxaloacetic transaminase 1, soluble | -4.391 | enzyme | Cytoplasm |
| SLC2A4: solute carrier family 2 (facilitated glucose transporter), member 4 | -4.429 | transporter | Plasma Membrane |
| SMAD6: SMAD family member 6 | -4.432 | transcription regulator | Nucleus |
| MT1A: metallothionein 1A | -4.438 | other | Cytoplasm |
| C14orf180: chromosome 14 open reading frame 180 | -4.486 | other | Plasma Membrane |
| IRX2: iroquois homeobox 2 | -4.5 | transcription regulator | Nucleus |
| MYOM2: myomesin 2 | -4.503 | other | Cytoplasm |
| ZNF385B: zinc finger protein 385B | -4.511 | other | Nucleus |
| COX7A1: cytochrome c oxidase subunit VIIa polypeptide 1 (muscle) | -4.559 | enzyme | Cytoplasm |
| STRIP2: striatin interacting protein 2 | -4.561 | other | Cytoplasm |
| GADD45G: growth arrest and DNA-damage-inducible, gamma | -4.757 | other | Nucleus |
| FREM2: FRAS1 related extracellular matrix protein 2 | -4.776 | other | Extracellular Space |
| SLC6A8: solute carrier family 6 (neurotransmitter transporter), member 8 | -4.815 | transporter | Cytoplasm |
| POPDC2: popeye domain containing 2 | -4.847 | other | Unknown |
| GTF2IRD1: GTF2I repeat domain containing 1 | -4.869 | transcription regulator | Nucleus |
| MPP3: membrane protein, palmitoylated 3 (MAGUK p55 subfamily member 3) | -4.923 | kinase | Plasma Membrane |
| TNNI1: troponin I type 1 (skeletal, slow) | -4.956 | other | Cytoplasm |
| FCN3: ficolin (collagen/fibrinogen domain containing) 3 | -4.962 | other | Extracellular Space |
| NKD2: naked cuticle homolog 2 (Drosophila) | -4.975 | other | Nucleus |
| SLC4A3: solute carrier family 4 (anion exchanger), member 3 | -5.03 | transporter | Plasma Membrane |
| MYH14: myosin, heavy chain 14, non-muscle | -5.06 | other | Extracellular Space |
| HRC: histidine rich calcium binding protein | -5.062 | other | Cytoplasm |
| CAMK2B: calcium/calmodulin-dependent protein kinase II beta | -5.087 | kinase | Cytoplasm |
| GPX3: glutathione peroxidase 3 (plasma) | -5.098 | enzyme | Extracellular Space |
| LMOD2: leiomodin 2 (cardiac) | -5.133 | other | Unknown |
| CDH19: cadherin 19, type 2 | -5.166 | other | Plasma Membrane |
| LPL: lipoprotein lipase | -5.196 | enzyme | Cytoplasm |
| ADAMTS4: ADAM metallopeptidase with thrombospondin type 1 motif, 4 | -5.257 | peptidase | Extracellular Space |
| CKB: creatine kinase, brain | -5.291 | kinase | Cytoplasm |
| LDLR: low density lipoprotein receptor | -5.305 | transporter | Plasma Membrane |
| SAA1: serum amyloid A1 | -5.31 | transporter | Extracellular Space |
| RPL3L: ribosomal protein L3-like | -5.396 | other | Cytoplasm |

| | | | |
|---|---|---|---|
| UNC45B: unc-45 homolog B (C. elegans) | -5.424 | other | Cytoplasm |
| LINC00881: long intergenic non-protein coding RNA 881 | -5.425 | other | Unknown |
| HSPB7: heat shock 27kDa protein family, member 7 (cardiovascular) | -5.462 | other | Cytoplasm |
| KLF15: Kruppel-like factor 15 | -5.465 | transcription regulator | Nucleus |
| B4GALNT3: beta-1,4-N-acetyl-galactosaminyl transferase 3 | -5.521 | enzyme | Unknown |
| ASB11: ankyrin repeat and SOCS box containing 11, E3 ubiquitin protein ligase | -5.522 | transcription regulator | Nucleus |
| KLHL40: kelch-like family member 40 | -5.545 | other | Unknown |
| SLC25A4: solute carrier family 25 (mitochondrial carrier; adenine nucleotide translocator), me | -5.556 | transporter | Cytoplasm |
| PTX3: pentraxin 3, long | -5.562 | other | Extracellular Space |
| ELF3: E74-like factor 3 (ets domain transcription factor, epithelial-specific ) | -5.629 | transcription regulator | Nucleus |
| MYO18B: myosin XVIIIB | -5.694 | other | Cytoplasm |
| PPARGC1A: peroxisome proliferator-activated receptor gamma, coactivator 1 alpha | -5.725 | transcription regulator | Nucleus |
| ANKRD2: ankyrin repeat domain 2 (stretch responsive muscle) | -5.738 | transcription regulator | Nucleus |
| IRX3: iroquois homeobox 3 | -5.78 | transcription regulator | Nucleus |
| FLNC: filamin C, gamma | -5.981 | other | Cytoplasm |
| PCDH20: protocadherin 20 | -6.075 | other | Unknown |
| TAS2R43: taste receptor, type 2, member 43 | -6.267 | G-protein coupled recepto | Plasma Membrane |
| LINC00473: long intergenic non-protein coding RNA 473 | -6.397 | other | Unknown |
| CYP1A1: cytochrome P450, family 1, subfamily A, polypeptide 1 | -6.447 | enzyme | Cytoplasm |
| PRSS45: protease, serine, 45 | -6.487 | peptidase | Unknown |
| MGST1: microsomal glutathione S-transferase 1 | -6.612 | enzyme | Cytoplasm |
| TRIM55: tripartite motif containing 55 | -6.908 | other | Cytoplasm |
| HPR: haptoglobin-related protein | -6.981 | peptidase | Extracellular Space |
| CSRP3: cysteine and glycine-rich protein 3 (cardiac LIM protein) | -7.522 | other | Nucleus |
| CACNA1C-IT1: CACNA1C intronic transcript 1 (non-protein coding) | -7.536 | other | Unknown |
| TNNC1: troponin C type 1 (slow) | -7.615 | other | Cytoplasm |
| FASN: fatty acid synthase | -7.661 | enzyme | Cytoplasm |
| TBX20: T-box 20 | -7.835 | transcription regulator | Nucleus |
| TNNI3: troponin I type 3 (cardiac) | -7.934 | transporter | Cytoplasm |
| RRAD: Ras-related associated with diabetes | -8.014 | enzyme | Cytoplasm |
| NDRG4: NDRG family member 4 | -8.061 | other | Unknown |
| AZGP1: alpha-2-glycoprotein 1, zinc-binding | -8.082 | transporter | Extracellular Space |
| NPPB: natriuretic peptide B | -8.346 | other | Extracellular Space |
| EEF1A2: eukaryotic translation elongation factor 1 alpha 2 | -8.464 | translation regulator | Cytoplasm |
| XIRP2: xin actin-binding repeat containing 2 | -8.473 | other | Unknown |
| JPH1: junctophilin 1 | -8.831 | other | Plasma Membrane |
| SERPINA3: serpin peptidase inhibitor, clade A (alpha-1 antiproteinase, antitrypsin), member [illegible] | -9.146 | other | Extracellular Space |
| ABRA: actin-binding Rho activating protein | -9.338 | transcription regulator | Cytoplasm |

| | | | |
|---|---|---|---|
| MYPN: myopalladin | -9.537 | other | Cytoplasm |
| IL1RL1: interleukin 1 receptor-like 1 | -9.603 | transmembrane receptor | Plasma Membrane |
| CKM: creatine kinase, muscle | -10.093 | kinase | Cytoplasm |
| GATA4: GATA binding protein 4 | -10.105 | transcription regulator | Nucleus |
| ANKRD1: ankyrin repeat domain 1 (cardiac muscle) | -10.173 | transcription regulator | Cytoplasm |
| TRIM63: tripartite motif containing 63, E3 ubiquitin protein ligase | -10.22 | enzyme | Nucleus |
| MYL3: myosin, light chain 3, alkali; ventricular, skeletal, slow | -12.005 | other | Cytoplasm |
| ZFP57: ZFP57 zinc finger protein | -12.047 | transcription regulator | Nucleus |
| NRAP: nebulin-related anchoring protein | -12.763 | other | Cytoplasm |
| MYH6: myosin, heavy chain 6, cardiac muscle, alpha | -14.387 | enzyme | Cytoplasm |
| XIRP1: xin actin-binding repeat containing 1 | -18.38 | other | Plasma Membrane |
| LOC285556: uncharacterized LOC285556 | -20.47 | other | Unknown |

© 2000-2014 Ingenuity Systems, Inc. All rights reserved.

**Supplemental Table 3. Dysregulated molecular pathways (8 KD cases vs 7 controls)**

| Pathways | DEGs | pValue |
|---|---|---|
| Primary Immunodeficiency Signaling | 26 | 5.65348E-21 |
| Communication between Innate and Adaptive Immune Cells | 30 | 1.08186E-17 |
| iCOS-iCOSL Signaling in T Helper Cells | 32 | 3.1186E-17 |
| Altered T Cell and B Cell Signaling in Rheumatoid Arthritis | 29 | 3.83575E-17 |
| B Cell Development | 17 | 3.62354E-14 |
| CD28 Signaling in T Helper Cells | 28 | 1.34919E-12 |
| Role of NFAT in Regulation of the Immune Response | 33 | 6.43132E-12 |
| PKCθ Signaling in T Lymphocytes | 27 | 8.5034E-12 |
| Autoimmune Thyroid Disease Signaling | 17 | 5.10052E-11 |
| Hematopoiesis from Pluripotent Stem Cells | 17 | 1.05849E-10 |
| T Cell Receptor Signaling | 23 | 1.37973E-10 |
| Antigen Presentation Pathway | 14 | 6.88573E-10 |
| Calcium-induced T Lymphocyte Apoptosis | 18 | 7.0292E-10 |
| Crosstalk between Dendritic Cells and Natural Killer Cells | 21 | 9.60653E-10 |
| Systemic Lupus Erythematosus Signaling | 34 | 1.80005E-09 |
| Allograft Rejection Signaling | 20 | 3.15616E-09 |
| Graft-versus-Host Disease Signaling | 15 | 3.7009E-09 |
| T Helper Cell Differentiation | 18 | 4.41087E-09 |
| CTLA4 Signaling in Cytotoxic T Lymphocytes | 20 | 4.84116E-09 |
| Nur77 Signaling in T Lymphocytes | 16 | 6.42304E-09 |
| Dendritic Cell Maturation | 29 | 8.48813E-09 |
| Agranulocyte Adhesion and Diapedesis | 29 | 2.965E-08 |
| OX40 Signaling Pathway | 19 | 3.47848E-08 |
| Natural Killer Cell Signaling | 21 | 5.32136E-08 |
| Hepatic Fibrosis / Hepatic Stellate Cell Activation | 29 | 7.52606E-08 |
| Phospholipase C Signaling | 32 | 1.55327E-07 |
| TREM1 Signaling | 16 | 4.20503E-07 |
| Cdc42 Signaling | 25 | 4.25915E-07 |
| Leukocyte Extravasation Signaling | 27 | 9.94328E-07 |
| Role of Macrophages, Fibroblasts and Endothelial Cells in Rheumatoid Arthritis | 35 | 1.01207E-06 |
| LXR/RXR Activation | 20 | 1.2367E-06 |
| B Cell Receptor Signaling | 24 | 3.92104E-06 |
| Granulocyte Adhesion and Diapedesis | 24 | 4.33367E-06 |
| Atherosclerosis Signaling | 19 | 6.35784E-06 |
| IL-4 Signaling | 14 | 1.34785E-05 |
| Type I Diabetes Mellitus Signaling | 17 | 1.91582E-05 |
| NF-κB Signaling | 22 | 2.97739E-05 |
| Role of Pattern Recognition Receptors in Recognition of Bacteria and Viruses | 18 | 3.66144E-05 |
| TR/RXR Activation | 14 | 4.98809E-05 |
| CCR5 Signaling in Macrophages | 12 | 9.89103E-05 |
| Role of JAK1 and JAK3 in γc Cytokine Signaling | 11 | 0.0002 |
| IL-8 Signaling | 21 | 0.0002 |
| Toll-like Receptor Signaling | 12 | 0.0002 |
| Creatine-phosphate Biosynthesis | 3 | 0.0004 |
| Tec Kinase Signaling | 18 | 0.0006 |
| Eicosanoid Signaling | 10 | 0.0008 |
| IL-2 Signaling | 9 | 0.0009 |
| G-Protein Coupled Receptor Signaling | 24 | 0.001 |
| MSP-RON Signaling Pathway | 8 | 0.001 |
| Role of MAPK Signaling in the Pathogenesis of Influenza | 10 | 0.001 |

| | | |
|---|---|---|
| Phospholipases | 9 | 0.001 |
| Fcγ Receptor-mediated Phagocytosis in Macrophages and Monocytes | 12 | 0.002 |
| Fc Epsilon RI Signaling | 13 | 0.002 |
| NF-κB Activation by Viruses | 10 | 0.002 |
| Cytotoxic T Lymphocyte-mediated Apoptosis of Target Cells | 6 | 0.004 |
| Gα12/13 Signaling | 13 | 0.004 |
| Reelin Signaling in Neurons | 10 | 0.004 |
| Signaling by Rho Family GTPases | 21 | 0.005 |
| Role of Osteoblasts, Osteoclasts and Chondrocytes in Rheumatoid Arthritis | 20 | 0.005 |
| cAMP-mediated signaling | 20 | 0.005 |
| Sperm Motility | 13 | 0.005 |
| IL-10 Signaling | 9 | 0.005 |
| IL-9 Signaling | 6 | 0.005 |
| Tryptophan Degradation to 2-amino-3-carboxymuconate Semialdehyde | 3 | 0.005 |
| Triacylglycerol Biosynthesis | 6 | 0.006 |
| Triacylglycerol Degradation | 5 | 0.006 |
| Chemokine Signaling | 9 | 0.007 |
| Caveolar-mediated Endocytosis Signaling | 9 | 0.007 |
| Pathogenesis of Multiple Sclerosis | 3 | 0.007 |
| FXR/RXR Activation | 13 | 0.008 |
| ILK Signaling | 17 | 0.008 |
| PI3K Signaling in B Lymphocytes | 13 | 0.009 |
| Actin Cytoskeleton Signaling | 19 | 0.009 |
| GM-CSF Signaling | 8 | 0.009 |
| Paxillin Signaling | 11 | 0.01 |
| Virus Entry via Endocytic Pathways | 10 | 0.01 |
| FcγRIIB Signaling in B Lymphocytes | 6 | 0.01 |
| IL-15 Signaling | 8 | 0.01 |
| Cardiomyocyte Differentiation via BMP Receptors | 4 | 0.01 |
| Antioxidant Action of Vitamin C | 10 | 0.02 |
| Endothelin-1 Signaling | 15 | 0.02 |
| MIF-mediated Glucocorticoid Regulation | 5 | 0.02 |
| Oleate Biosynthesis II (Animals) | 3 | 0.02 |
| Retinol Biosynthesis | 5 | 0.02 |
| Calcium Signaling | 15 | 0.02 |
| RANK Signaling in Osteoclasts | 9 | 0.03 |
| Production of Nitric Oxide and Reactive Oxygen Species in Macrophages | 15 | 0.03 |
| Human Embryonic Stem Cell Pluripotency | 12 | 0.03 |
| Glycerol Degradation I | 2 | 0.03 |
| Factors Promoting Cardiogenesis in Vertebrates | 9 | 0.03 |
| NAD biosynthesis II (from tryptophan) | 3 | 0.03 |
| CDP-diacylglycerol Biosynthesis I | 3 | 0.03 |
| Acute Phase Response Signaling | 14 | 0.03 |
| fMLP Signaling in Neutrophils | 10 | 0.03 |
| Protein Ubiquitination Pathway | 6 | 0.04 |
| Regulation of IL-2 Expression in Activated and Anergic T Lymphocytes | 8 | 0.04 |
| CD40 Signaling | 7 | 0.04 |
| SAPK/JNK Signaling | 9 | 0.04 |
| Docosahexaenoic Acid (DHA) Signaling | 5 | 0.04 |
| B Cell Activating Factor Signaling | 5 | 0.04 |
| Lymphotoxin β Receptor Signaling | 6 | 0.04 |
| Role of IL-17A in Arthritis | 6 | 0.04 |

| | | |
|---|---|---|
| CDK5 Signaling | 1 | 0.05 |
| MIF Regulation of Innate Immunity | 5 | 0.05 |
| Phosphatidylglycerol Biosynthesis II (Non-plastidic) | 3 | 0.05 |
| γ-linolenate Biosynthesis II (Animals) | 3 | 0.05 |
| Dopamine-DARPP32 Feedback in cAMP Signaling | 3 | 0.05 |
| Melatonin Degradation III | 1 | 0.05 |

© 2000-2014 Ingenuity Systems, Inc. All rights reserved.

**Supplemental Table 4.** Differential expression of genes involved in antigen presentation and dendritic cell function in KD compared to control coronary arteries by real-time reverse transcriptase PCR assays

| Gene | Fold Change (95% CI) | Adjusted *p*-value |
|---|---|---|
| CD74 | 11.2 (3.3-37.9) | 0.001 |
| CD226 | 7.9 (2.3-26.9) | 0.004 |
| CD69 | 6.9 (2.0-23.5) | 0.005 |
| IL18 | 6.3 (1.9-21.6) | 0.006 |
| NLRC5 | 5.3 (1.5-17.9) | 0.010 |
| HLA-F | 4.2 (1.2-14.4) | 0.022 |

**Supplemental Table 5. Extracellular genes dysregulated in 8 KD coronary arteries compared with 7 childhood control coronary arteries**

| Gene | Fold change | Molecular Function |
|---|---|---|
| CXCL9: chemokine (C-X-C motif) ligand 9 | 48.0 | cytokine |
| IGHG1: immunoglobulin heavy constant gamma 1 (G1m marker) | 45.0 | other |
| IGJ: immunoglobulin J polypeptide, linker protein for immunoglobulin alpha and mu polypeptides | 31.3 | other |
| CXCL13: chemokine (C-X-C motif) ligand 13 | 21.8 | cytokine |
| ADAMDEC1: ADAM-like, decysin 1 | 20.5 | peptidase |
| IGHG3: immunoglobulin heavy constant gamma 3 (G3m marker) | 17.0 | other |
| IGKV3-20: immunoglobulin kappa variable 3-20 | 16.0 | other |
| IGHA1: immunoglobulin heavy constant alpha 1 | 12.8 | other |
| IGHG4: immunoglobulin heavy constant gamma 4 (G4m marker) | 12.5 | other |
| IGLC3: immunoglobulin lambda constant 3 (Kern-Oz+ marker) | 11.9 | other |
| IGKV1-5: immunoglobulin kappa variable 1-5 | 11.5 | other |
| COMP: cartilage oligomeric matrix protein | 11.4 | other |
| CCL18: chemokine (C-C motif) ligand 18 (pulmonary and activation-regulated) | 10.6 | cytokine |
| ACAN: aggrecan | 10.4 | other |
| IGLC2: immunoglobulin lambda constant 2 (Kern-Oz- marker) | 10.3 | other |
| NELL2: NEL-like 2 (chicken) | 8.8 | other |
| LYZ: lysozyme | 8.4 | enzyme |
| MXRA5: matrix-remodelling associated 5 | 8.1 | other |
| CXCL14: chemokine (C-X-C motif) ligand 14 | 8.1 | cytokine |
| COL11A1: collagen, type XI, alpha 1 | 7.5 | other |
| IGKC: immunoglobulin kappa constant | 7.3 | other |
| RNASE6: ribonuclease, RNase A family, k6 | 6.9 | enzyme |
| POSTN: periostin, osteoblast specific factor | 6.5 | other |
| CCL5: chemokine (C-C motif) ligand 5 | 6.5 | cytokine |
| IGKV4-1: immunoglobulin kappa variable 4-1 | 6.4 | other |
| PRSS35: protease, serine, 35 | 6.4 | peptidase |
| SCG2: secretogranin II | 6.2 | cytokine |
| EGFL6: EGF-like-domain, multiple 6 | 6.2 | other |
| AOAH: acyloxyacyl hydrolase (neutrophil) | 6.0 | enzyme |
| PLA2G7: phospholipase A2, group VII (platelet-activating factor acetylhydrolase, plasma) | 5.9 | enzyme |
| XCL1: chemokine (C motif) ligand 1 | 5.4 | cytokine |
| FAM46C: family with sequence similarity 46, member C | 5.3 | other |

| | | |
|---|---|---|
| IGHA2: immunoglobulin heavy constant alpha 2 (A2m marker) | 5.0 | other |
| IGHD: immunoglobulin heavy constant delta | 4.8 | other |
| SAMD9L: sterile alpha motif domain containing 9-like | 4.7 | other |
| CECR1: cat eye syndrome chromosome region, candidate 1 | 4.7 | enzyme |
| FCN1: ficolin (collagen/fibrinogen domain containing) 1 | 4.7 | other |
| LTBP2: latent transforming growth factor beta binding protein 2 | 4.7 | other |
| OLFML2B: olfactomedin-like 2B | 4.5 | other |
| DMP1: dentin matrix acidic phosphoprotein 1 | 4.4 | other |
| TNFSF13B: tumor necrosis factor (ligand) superfamily, member 13b | 4.2 | cytokine |
| BTN3A1: butyrophilin, subfamily 3, member A1 | 4.1 | other |
| ERMN: ermin, ERM-like protein | 4.1 | other |
| C16orf54: chromosome 16 open reading frame 54 | 4.0 | other |
| ASPN: asporin | 4.0 | other |
| TNFSF11: tumor necrosis factor (ligand) superfamily, member 11 | 4.0 | cytokine |
| FASLG: Fas ligand (TNF superfamily, member 6) | 3.9 | cytokine |
| COL22A1: collagen, type XXII, alpha 1 | 3.6 | other |
| ADAMTS14: ADAM metallopeptidase with thrombospondin type 1 motif, 14 | 3.6 | peptidase |
| SLITRK4: SLIT and NTRK-like family, member 4 | 3.5 | other |
| NOG: noggin | 3.5 | growth factor |
| GLT1D1: glycosyltransferase 1 domain containing 1 | 3.5 | enzyme |
| IL24: interleukin 24 | 3.4 | cytokine |
| FGL2: fibrinogen-like 2 | 3.4 | peptidase |
| IL18: interleukin 18 | 3.4 | cytokine |
| CXCL16: chemokine (C-X-C motif) ligand 16 | 3.4 | cytokine |
| AIM1: absent in melanoma 1 | 3.4 | other |
| FDCSP: follicular dendritic cell secreted protein | 3.4 | other |
| MZB1: marginal zone B and B1 cell-specific protein | 3.4 | other |
| TEX15: testis expressed 15 | 3.4 | other |
| PLXDC2: plexin domain containing 2 | 3.3 | other |
| BANK1: B-cell scaffold protein with ankyrin repeats 1 | 3.2 | other |
| DEF6: differentially expressed in FDCP 6 homolog (mouse) | 3.2 | other |
| SPINK1: serine peptidase inhibitor, Kazal type 1 | 3.2 | other |
| BGN: biglycan | 3.1 | other |
| OSM: oncostatin M | 3.0 | cytokine |

| | | |
|---|---|---|
| CTGF: connective tissue growth factor | 2.9 | growth factor |
| IL1RN: interleukin 1 receptor antagonist | 2.8 | cytokine |
| COL8A1: collagen, type VIII, alpha 1 | 2.8 | other |
| CCL22: chemokine (C-C motif) ligand 22 | 2.8 | cytokine |
| SMOC2: SPARC related modular calcium binding 2 | 2.8 | other |
| ABCA13: ATP-binding cassette, sub-family A (ABC1), member 13 | 2.8 | transporter |
| LIPM: lipase, family member M | 2.7 | enzyme |
| TDGF1: teratocarcinoma-derived growth factor 1 | 2.7 | growth factor |
| PZP: pregnancy-zone protein | 2.6 | other |
| EDIL3: EGF-like repeats and discoidin I-like domains 3 | 2.6 | other |
| SPOCK3: sparc/osteonectin, cwcv and kazal-like domains proteoglycan (testican) 3 | 2.6 | other |
| CA6: carbonic anhydrase VI | 2.6 | enzyme |
| LPO: lactoperoxidase | 2.5 | enzyme |
| VNN3: vanin 3 | 2.5 | enzyme |
| LTB: lymphotoxin beta (TNF superfamily, member 3) | 2.3 | cytokine |
| DEFA4: defensin, alpha 4, corticostatin | 2.3 | other |
| APELA: apelin receptor early endogenous ligand | 2.3 | other |
| CPXM2: carboxypeptidase X (M14 family), member 2 | 2.2 | peptidase |
| FGF20: fibroblast growth factor 20 | 2.2 | growth factor |
| OGN: osteoglycin | 2.0 | growth factor |
| PAPLN: papilin, proteoglycan-like sulfated glycoprotein | 2.0 | other |
| PI16: peptidase inhibitor 16 | 1.9 | other |
| IBSP: integrin-binding sialoprotein | 1.7 | other |
| EREG: epiregulin | 1.7 | growth factor |
| OLFM4: olfactomedin 4 | 1.6 | other |
| EPPIN: epididymal peptidase inhibitor | 1.6 | other |
| F13B: coagulation factor XIII, B polypeptide | 1.6 | enzyme |
| PGC: progastricsin (pepsinogen C) | -1.5 | peptidase |
| CHI3L2: chitinase 3-like 2 | -1.6 | enzyme |
| BMP10: bone morphogenetic protein 10 | -1.6 | growth factor |
| BMP3: bone morphogenetic protein 3 | -1.6 | growth factor |
| PLA2G1B: phospholipase A2, group IB (pancreas) | -1.7 | enzyme |
| ADIPOQ: adiponectin, C1Q and collagen domain containing | -1.7 | other |
| CCL2: chemokine (C-C motif) ligand 2 | -1.8 | cytokine |

| | | |
|---|---|---|
| LAMB3: laminin, beta 3 | -1.8 | transporter |
| PCSK9: proprotein convertase subtilisin/kexin type 9 | -1.8 | peptidase |
| LEP: leptin | -1.9 | growth factor |
| CPB2: carboxypeptidase B2 (plasma) | -1.9 | peptidase |
| LGALS12: lectin, galactoside-binding, soluble, 12 | -2.0 | other |
| NMB: neuromedin B | -2.0 | other |
| LIPH: lipase, member H | -2.1 | enzyme |
| LIPG: lipase, endothelial | -2.2 | enzyme |
| AGR2: anterior gradient 2 | -2.2 | other |
| NAPSA: napsin A aspartic peptidase | -2.3 | peptidase |
| SFTPC: surfactant protein C | -2.3 | other |
| RBP4: retinol binding protein 4, plasma | -2.3 | transporter |
| ADAMTS19: ADAM metallopeptidase with thrombospondin type 1 motif, 19 | -2.4 | peptidase |
| SFTPB: surfactant protein B | -2.4 | other |
| ADAMTS15: ADAM metallopeptidase with thrombospondin type 1 motif, 15 | -2.4 | peptidase |
| SHH: sonic hedgehog | -2.5 | peptidase |
| TF: transferrin | -2.5 | transporter |
| CSF3: colony stimulating factor 3 (granulocyte) | -2.6 | cytokine |
| TFPI2: tissue factor pathway inhibitor 2 | -2.6 | other |
| ARTN: artemin | -2.6 | growth factor |
| COL4A4: collagen, type IV, alpha 4 | -2.7 | other |
| F11: coagulation factor XI | -2.7 | peptidase |
| WIF1: WNT inhibitory factor 1 | -2.8 | other |
| MSLN: mesothelin | -2.9 | other |
| SAA2: serum amyloid A2 | -2.9 | other |
| TIMP4: TIMP metallopeptidase inhibitor 4 | -2.9 | other |
| WISP2: WNT1 inducible signaling pathway protein 2 | -3.0 | growth factor |
| HAMP: hepcidin antimicrobial peptide | -3.0 | other |
| PAPPA: pregnancy-associated plasma protein A, pappalysin 1 | -3.0 | peptidase |
| HP: haptoglobin | -3.0 | peptidase |
| ADAMTS9: ADAM metallopeptidase with thrombospondin type 1 motif, 9 | -3.1 | peptidase |
| C1QTNF1: C1q and tumor necrosis factor related protein 1 | -3.2 | other |
| FAM213A: family with sequence similarity 213, member A | -3.2 | other |
| COL4A3: collagen, type IV, alpha 3 (Goodpasture antigen) | -3.2 | other |

| | | |
|---|---|---|
| ADAMTS8: ADAM metallopeptidase with thrombospondin type 1 motif, 8 | -3.3 | peptidase |
| SFTPA2: surfactant protein A2 | -3.3 | other |
| FRAS1: Fraser syndrome 1 | -3.4 | other |
| SFTPA1: surfactant protein A1 | -3.4 | transporter |
| CFD: complement factor D (adipsin) | -3.5 | peptidase |
| SFTPD: surfactant protein D | -4.0 | other |
| MOG: myelin oligodendrocyte glycoprotein | -4.2 | other |
| CORO6: coronin 6 | -4.2 | other |
| TAC1: tachykinin, precursor 1 | -4.2 | other |
| CHI3L1: chitinase 3-like 1 (cartilage glycoprotein-39) | -4.3 | enzyme |
| FREM2: FRAS1 related extracellular matrix protein 2 | -4.8 | other |
| FCN3: ficolin (collagen/fibrinogen domain containing) 3 | -5.0 | other |
| MYH14: myosin, heavy chain 14, non-muscle | -5.1 | other |
| GPX3: glutathione peroxidase 3 (plasma) | -5.1 | enzyme |
| ADAMTS4: ADAM metallopeptidase with thrombospondin type 1 motif, 4 | -5.3 | peptidase |
| SAA1: serum amyloid A1 | -5.3 | transporter |
| PTX3: pentraxin 3, long | -5.6 | other |
| HPR: haptoglobin-related protein | -7.0 | peptidase |
| AZGP1: alpha-2-glycoprotein 1, zinc-binding | -8.1 | transporter |
| NPPB: natriuretic peptide B | -8.3 | other |
| SERPINA3: serpin peptidase inhibitor, clade A (alpha-1 antiproteinase, antitrypsin), member 3 | -9.1 | other |

© 2000-2014 Ingenuity Systems, Inc. All rights reserved.

**Supplemental Figure Legend.**

Supplemental Figure 1. Principal components analysis of all genes in 8 KD (4 treated and 4 untreated) and 7 childhood control coronary artery tissues demonstrates that gene expression of untreated (red dots) and treated (black dots) KD patients are not distinguishable. Ellipses show the 80% confidence interval for gene expression in KD (red dashed line) and control (blue dashed line) coronary arteries. As expected, there is partial overlap of KD and control gene expression, although gene expression in most of the KD tissue samples falls outside the control gene expression ellipse.

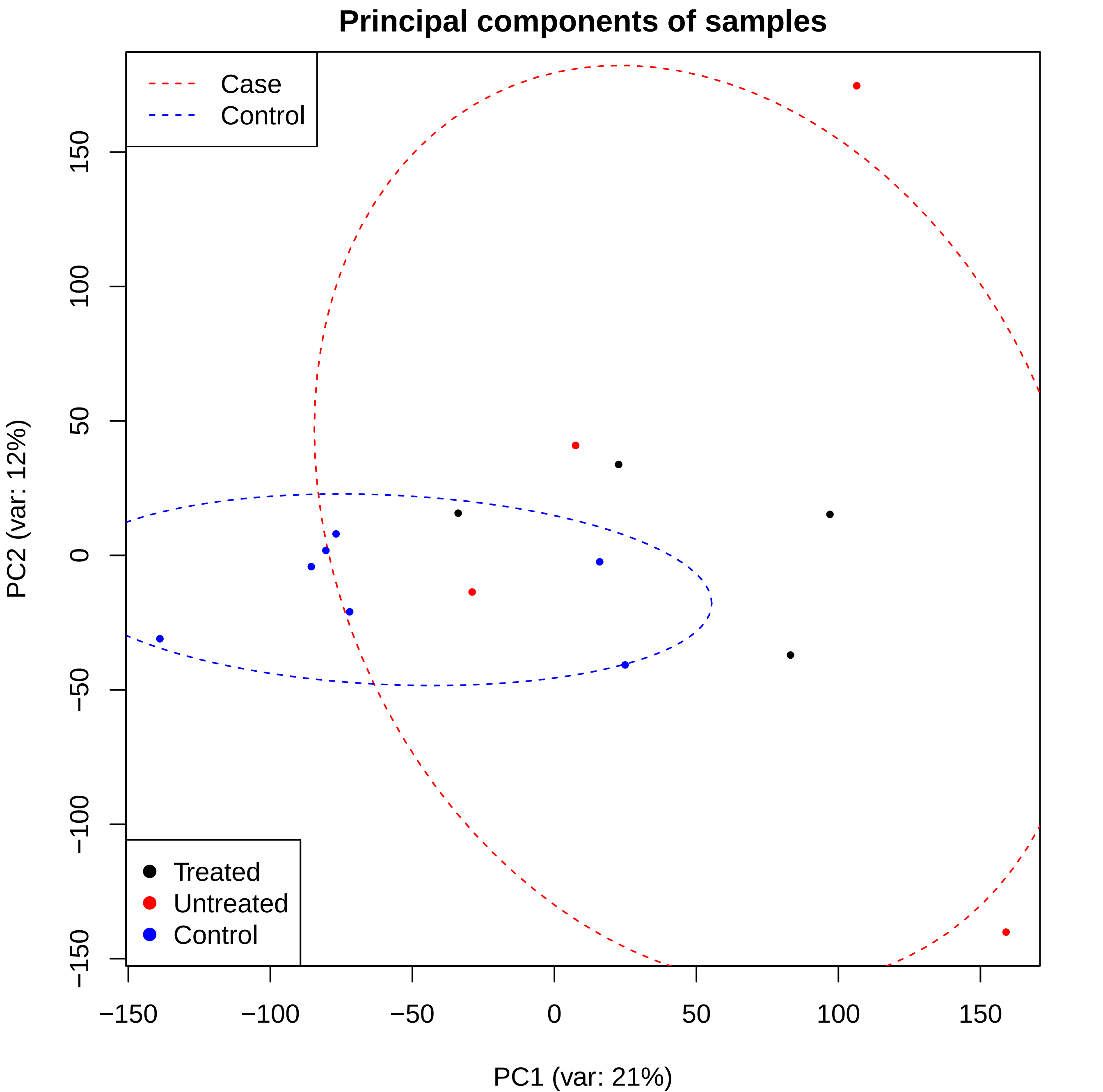
Principal components of samples
Case
Control
Treated
Untreated
Control
PC1 (var: 21%)
PC2 (var: 12%)
−150
−100
−50
0
50
100
150
